# Comparison of reversed-phase, hydrophilic interaction, and porous graphitic carbon chromatography columns for an untargeted toxicometabolomics study in pooled human liver microsomes, rat urine, and rat plasma

**DOI:** 10.1007/s11306-024-02115-0

**Published:** 2024-04-30

**Authors:** Selina Hemmer, Sascha K. Manier, Lea Wagmann, Markus R. Meyer

**Affiliations:** Department of Experimental and Clinical Toxicology, Institute of Experimental and Clinical Pharmacology and Toxicology, Center for Molecular Signaling (PZMS), Saarland University, Homburg, Germany

**Keywords:** Untargeted metabolomics, LC-HRMS, Reversed-phase columns, Hydrophilic interaction chromatography columns, Quality assurance

## Abstract

**Introduction:**

Untargeted metabolomics studies are expected to cover a wide range of compound classes with high chemical diversity and complexity. Thus, optimizing (pre-)analytical parameters such as the analytical liquid chromatography (LC) column is crucial and the selection of the column depends primarily on the study purpose.

**Objectives:**

The current investigation aimed to compare six different analytical columns. First, by comparing the chromatographic resolution of selected compounds. Second, on the outcome of an untargeted toxicometabolomics study using pooled human liver microsomes (pHLM), rat plasma, and rat urine as matrices.

**Methods:**

Separation and analysis were performed using three different reversed-phase (Phenyl-Hexyl, BEH C_18_, and Gold C_18_), two hydrophilic interaction chromatography (HILIC) (ammonium-sulfonic acid and sulfobetaine), and one porous graphitic carbon (PGC) columns coupled to high-resolution mass spectrometry (HRMS). Their impact was evaluated based on the column performance and the size of feature count, amongst others.

**Results:**

All three reversed-phase columns showed a similar performance, whereas the PGC column was superior to both HILIC columns at least for polar compounds. Comparing the size of feature count across all datasets, most features were detected using the Phenyl-Hexyl or sulfobetaine column. Considering the matrices, most significant features were detected in urine and pHLM after using the sulfobetaine and in plasma after using the ammonium-sulfonic acid column.

**Conclusion:**

The results underline that the outcome of this untargeted toxicometabolomic study LC-HRMS metabolomic study was highly influenced by the analytical column, with the Phenyl-Hexyl or sulfobetaine column being the most suitable. However, column selection may also depend on the investigated compounds as well as on the investigated matrix.

**Graphical abstract:**

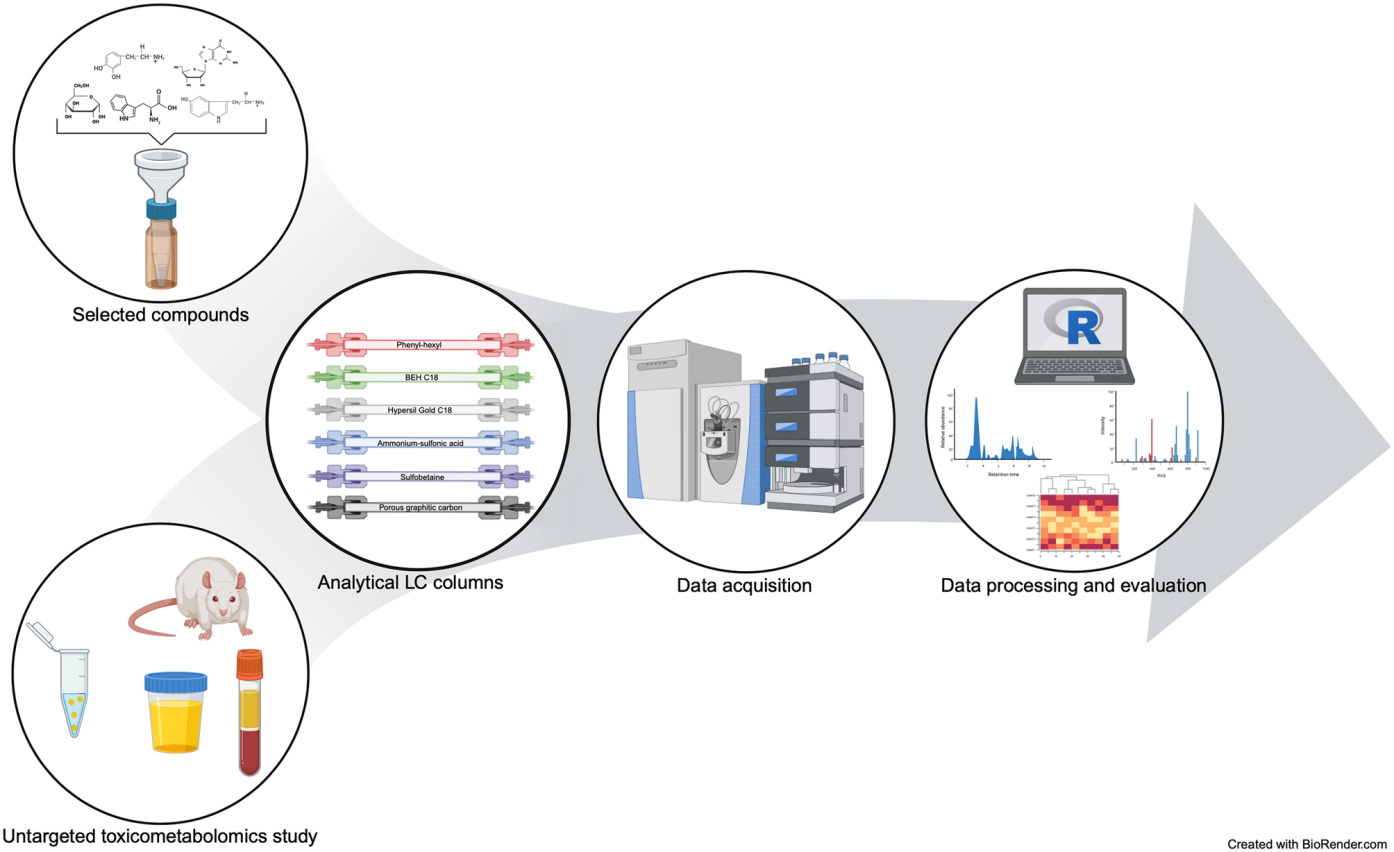

**Supplementary Information:**

The online version contains supplementary material available at 10.1007/s11306-024-02115-0.

## Introduction

Metabolomics studies can in general be divided into untargeted and targeted approaches. Whereas targeted metabolomics aims to detect and quantify specific metabolites of known structures and pathways, untargeted metabolomic studies, as a global approach, aim to detect as many metabolites as possible (Agin et al., 2016; Barnes et al., 2016a). Due to several advantages, liquid chromatography (LC) and mass spectrometry (MS) are meanwhile the major techniques used in metabolomics (Naz et al., 2014; Yao et al., 2019). The impact of LC is mainly influenced by the used stationary phase amongst others (Harrieder et al., 2022; Liu & Locasale, 2017). While normal- or hydrophilic interaction-phase chromatography (HILIC) columns are often used for retention of polar molecules such as amino acids or sugars, reversed-phase (RP) columns are used for non to mid polar molecules such as fatty acids or lipids. Thus, a broad range of compounds can be covered by using both types. However, as several stationary HILIC and RP phases are available, their choice is crucial, which was already discussed extensively elsewhere (Diamantidou et al., 2023; Elmsjo et al., 2018; Si-Hung et al., 2017; Sonnenberg et al., 2019; Wernisch & Pennathur, 2016). Not only the different stationary phases but also the geometry and particle size of columns can affect the outcome of metabolomic studies.

Most of the published studies on analytical column comparison are within the field of targeted metabolomics, investigated metabolite libraries with and without matrix, or developed a scoring approach for the comparison of different column types. To date, there are only a few studies available that did a column comparison within the field of untargeted metabolomics. One main issue in untargeted analysis are the heterogenous physicochemical properties of analytes, which are often even unknown beforehand. Thus, a more universal separation (and detection) system should be used (Harrieder et al., 2022; van de Velde et al., 2020). Multiple chromatographic methods are often used to enable a broad analyte coverage (Barnes et al., 2016a; Harrieder et al., 2022). Additionally, to ensure correct interpretation of differences in specific metabolites and for appropriate biological interpretation, a reliable and suitable overall approach is required, which should include the selection of the most appropriate column (Naz et al., 2014).

Therefore, the aim of this study was to compare of six different stationary phases, three reversed-phase, two hydrophilic interaction, and one porous graphitic carbon phase. First, by comparing the chromatographic resolution of selected compounds. Second, their impact on the outcome of an untargeted toxicometabolomics study (Hemmer et al., 2022). The (toxico-)metabolome of three different biological matrices should be investigated after exposure to the synthetic cathinone PCYP (2-cyclohexyl-1-phenyl-2-(1-pyrrolidinyl)-ethanone) as model compound. The study should statistically evaluate analytical columns based on their performance (chromatographic resolution of analytes) the number and quality of detected features after untargeted high-resolution mass spectrometry (HRMS) analysis. Finally, the study should statistically show, which combination of columns may be best suitable for which matrix in future studies.

## Experimental section

### Material and chemicals

PCYP hydrochloride was provided by the State Bureau of Criminal Investigation Schleswig–Holstein (EU project ADEBAR plus, Kiel, Germany) for research purposes. The chemical purity of > 93% and identity of the compound were verified by MS and nuclear magnetic resonance analysis.

25-HO Cholesterol, adenosine 5’ diphosphate, ammonium formate, ammonium acetate, arachidic acid, ascorbate, carnosine, chloroform, cholesteryl oleate, citrate, cortisone, creatinine, creatinine-d_3_, D-fructose, D-glucose, D-glucose-d_7_, D-ribose, dipotassium phosphate, dopamine, formic acid, glutamine, glutaminic acid, guanosine 5’ triphosphate, histamine, inosine, isocitrate dehydrogenase, isocitrate, kynurenine, lauric acid, lysin, magnesium chloride, maltose, NAD, noradrenalin, palmitic acid-d_31_, pregnenolone, proline, retinol, riboflavin, serotonin, spermidine, succinate, superoxide dismutase, threonine, tripotassium phosphate, tryptophane, and vitamine D2 were obtained from Merck (Darmstadt, Germany). Acetonitrile, ethanol, methanol (all LC–MS grade), and NADP-Na_2_ were from VWR (Darmstadt, Germany). L-Tryptophan-d_5_ was obtained from Alsachim (Illkirch-Graffenstaden, France). 1-Palmitoyl-d_9_-2-palmitoyl-*sn*-glycero-3-PC and prostaglandin-E3-d_9_ were from Cayman Chemical (Michigan, USA). Difluoroacetic acid (DFA) was obtained from Acros organics (Geel, Belgium). Water was purified with a Millipore filtration unit (18.2 Ω × cm water resistance). pHLM (20 mg microsomal protein × mL^−1^, 360 pmol total CYP/mg, 26 donors) were obtained from Corning (Amsterdam, The Netherlands). After delivery, pHLM were thawed at 37 °C, aliquoted, snap-frozen in liquid nitrogen, and stored at − 80 °C until use.

### Sample preparation and analysis of selected compounds

A total mixtures of 34 compounds consisting of different compound classes including nine amino acids, six biogenic amines, three carboxylic acids, one coenzyme, two fatty acids, one lipid, three nucleotides, three steroids, four sugars, and three vitamins were analyzed at a concentration level of 50 µg/mL using the six columns Phenyl-Hexyl, Gold C_18_ (Gold), BEH C_18_ (BEH), ammonium-sulfonic acid (Nucleodur), sulfobetaine (ZicHILIC), and porous graphitic carbon particle (PGC) (Table S1). Amino acids, carboxylic acids, biogenic amines, polyamines, nucleotides, coenzymes, and vitamins were dissolved in a water/methanol (95:5, v/v) mixture, sugars in Millipore water, and fatty acids, lipids, steroids, and hormones in chloroform/methanol (1:1, v/v) mixture.

### Sample handling of datasets.

Study design, sample collection, sample preparation for pooled human liver microsomes (pHLM), rat blood plasma, and rat urine were as described by Hemmer et al. (Hemmer et al., 2022).

pHLM incubations were performed using a final PCYP concentration of 0 (blank group) or 50 µM (PCYP group) and 1 mg protein mL^−1^ pHLM at 37 °C. The final incubation mixture also contained 90 mM phosphate buffer, 5 mM isocitrate, 5 mM Mg^2+^, 1.2 mM NADP^+^, 200 U/mL superoxide dismutase, and 0.5 U mL^−1^ isocitrate dehydrogenase. The reaction was stopped after 60 min by adding ice-cold acetonitrile and then centrifuged for 2 min at 18,407×*g*. For each group, 5 replicates were prepared. Pooled quality control samples (QC group) were prepared by transferring equal proportion of each sample into one MS vial. QC samples were used for optimization of peak-picking parameters, evaluating of column performance, and identification of significant features. QC samples, each sample of blank and PCYP group, were aliquoted into six separate MS vials and stored until use at − 80 °C. For each run with each column, one of the corresponding vials was retrieved from the freezer and measured. Thus, the same conditions were given for all columns.

In vivo studies were approved by an ethics committee (33/2019-Landesamt für Verbraucherschutz, Saarbrücken, Germany). Rat plasma and urine samples were collected from five control and five rats having a single dose of 2 mg/kg body weight PCYP administered.

Blood plasma samples were prepared as follows: an amount of 50 L plasma was transferred into a reaction tube and precipitated using 200 L of a mixture of methanol and ethanol (1:1, v/v). The mixture contained 48 M L-tryptophan-d_5_, 8.6 M creatinine-d_3_, 34.8 M palmitic acid-d_31_, and 53.4 M D-glucose-d_7_ as internal standard. Samples were shaken for 2 min at 2000 rpm and subsequently centrifuged at 21,130 × g and 2 °C for 30 min. A volume of 150 L of the supernatant was transferred into a new reaction tube and evaporated to dryness using a vacuum centrifuge at 1400 rpm and 24 °C for 20 min. The obtained residues were reconstituted in 50 L of a mixture of acetonitrile and methanol (70:30, v/v).

Urine samples were centrifuged at 13,523×g at 4 °C for 10 min. Volumes of 100 L of urine were transferred into reaction tubes and 400 L methanol, including 48 M L-tryptophan-d_5_, 8.6 M creatinine-d_3_, 34.8 M palmitic acid-d_31_, and 53.4 M D-glucose-d_7_ as internal standard, was added. Samples were cooled to − 20 °C for 20 min and then centrifuged at 13,523×*g* and 4 °C for 10 min. An amount of 350 L of the supernatant was transferred into a new reaction tube and evaporated to dryness using a vacuum centrifuge at 1400 rpm and 24 °C. The obtained residues were reconstituted in 50 L of a mixture of acetonitrile and methanol (70:30, v/v).

For each matrix and rat, three replicates were prepared and the corresponding 50 µL of them were added together, resulting in 150 µL per rat*.* Pooled quality control samples (QC group) were prepared for each matrix by transferring equal proportion of each sample into one MS vial. QC samples were used for optimization of peak-picking parameters, evaluating of column performance, and identification of significant features. QC samples, each sample of control rats and PCYP rats, were aliquoted into six separate MS vials and stored until use at − 80 °C. For each run with each column, one of the corresponding vials was retrieved from the freezer and measured. Thus, the same conditions were given for all columns.

### LC-HRMS apparatus

Analyses were performed using a Thermo Fisher Scientific (TF, Dreieich, Germany) Dionex UltiMate 3000 RS pump consisting of a degasser, a quaternary pump, and an UlitMate Autosampler, coupled to a TF Q Exactive Plus equipped with a heated electrospray ionization (HESI)-II source according to previous published studies (Hemmer et al., 2022; Manier et al., 2019a, 2019b). Performance of the columns and the mass spectrometer was tested before each batch using a test mixture described in the *Experimental Section* in the *Supplementary Information*. The used columns and their corresponding flow rates, gradients, and mobile phases are shown in Table 1. For preparation and cleaning of the injection system, isopropanol:water (90:10, *v/v*) was used. The following settings were used: wash volume, 100 µL; wash speed, 4000 nL/s; loop wash factor, 2. Column temperature for every analysis was set to 40 °C, maintained by a Dionex UltiMate 3000 RS analytical column heater. Injection volume was set to 1 µL for all samples, except for samples of the compound classes. HESI-II source conditions were as follow: ionization mode, positive or negative; sheath gas, 60 AU; auxiliary gas, 10 AU; sweep gas, 3 AU; spray voltage, 3.5 kV in positive and -4.0 kV in negative mode; heater temperature 320 °C; ion transfer capillary temperature, 320 °C; and S-lens RF level, 50.0. Mass spectrometry for untargeted metabolomics was performed according to a previously optimized workflow (Manier et al., 2019a, 2019b). The settings for full scan (FS) data acquisition were as follows: resolution 140,000 at *m/z* 200; microscan, 1; automatic gain control (AGC) target, 5e5; maximum injection time, 200 ms; scan range, *m/z* 50–750; spectrum data type; centroid. All study samples were analyzed in randomized order, to avoid potential analyte instability or instrument performance to confound data interpretation. Additionally, one QC injection was performed every five samples to monitor batch effects, as described by Wehrens et al. (Wehrens et al., 2016). Significant features were subsequently identified using parallel reaction monitoring (PRM). Settings for PRM data acquisition were as follow: resolution, 35,000 at *m/z* 200; microscans, 1; AGC target, 5e5; maximum injection time, 200 ms; isolation window, *m/z* 1.0; collisions energy (CE), 10, 20, 35, or 40 eV; spectrum data type, centroid. The inclusion list contained the monoisotopic masses of all significant features, and a time window of their retention time ± 60 s. The injection volume for the different mixture of compound classes was set to 2 µL and MS was carried out in full scan mode with subsequent data-dependent acquisition of MS^2^ (ddMS^2^) in positive and negative ionization mode. Following FS settings were used: resolution 35,000 at *m/z* 200; microscan, 1; AGC target, 5e4; maximum injection time, 120 ms; scan range, *m/z* 50–750. For ddMS^2^ mode the following settings were used: resolution 17,500 at *m/z* 200; microscan, 1; AGC target, 5e4; maximum injection time, 250 ms; scan range, *m/z* 50–750; isolation window, *m/z* 1.0; high collision dissociation cell with stepped normalized collision energy (NCE), 17.5, 35, and 52.5 eV; exclude isotopes, on; dynamic exclusion, 5 s; spectrum data type, profile. TF Xcalibur software version 3.0.63 was used for data handling.

**Table 1:** Overview of the used columns and their corresponding flow rates, gradients, and mobile phases

Column	Phenyl-Hexyl	Gold	BEH	Nucleodur	ZicHILIC	PGC
Chemistry	Phenyl/hexyl	C_18_	C_18_	Ammonium-sulfonic acid	Sulfobetaine	Porous graphitic carbon particle
Phase	Spherical, solid core, ultrapure silica	Spherical, fully porous, ultrapure silica	Ethylene bridged hybrid (BEH) particle technology	Fully porous particles	Fully porous particles	
Specification	Thermo Fisher Accucore Phenyl-Hexyl column	Thermo Fisher Hypersil Gold C_18_ column	Waters ACQUITY UPLC BEH C_18_ column	Macherey–Nagel HILIC Nucleodur column	Merck SeQuant ZIC HILIC	Merck PGC Supel™ Carbon LC
Dimensions	100 mm × 2.1 mm, 2.6 µm	100 mm × 2.1 mm, 1.9 µm	100 mm × 2.1 mm, 1.7 µm	125 mm × 3 mm, 3 µm	150 mm × 2.1 mm, 3.5 µm	150 mm × 2.1 mm, 2.7 µm
Flow rate	500 µL/min (1–10 min); 800 µL/min (10–13.5 min)			500 µL/min		
Gradient	0–1 min 99% A, 1–10 min to 1% A, 10–11.5 min hold 1% A, 11.5–13.5 min hold 99% A			0–1 min 2% A, 1–5 min to 20% A, 5–8.5 min to 60% A, 8.5–10 min hold 60% A, 10–12 min hold 2% A		0–1 min 99% A, 1–10 min to 1% A, 10–11.5 min hold 1% A, 11.5–13.5 min hold 99% A
Mobile phase	Eluent A: aqueous ammonium formate (2 mM), acetonitrile (1%, *v/v*) and formic acid (0.1%, *v/v*, pH 3) Eluent B: ammonium formate solution (2 mM) in acetonitrile:methanol (1:1, *v/v*), water (1%, *v/v*), and formic acid (0.1%, *v/v*)	Eluent A: 10 mM aqueous ammonium acetate containing acetonitrile (1%, *v/v*) and formic acid (0.1%, *v/v*, pH 3) Eluent B: acetonitrile containing formic acid (0.1%, *v/v*)		Eluent A: aqueous ammonium acetate (200 mM) Eluent B: acetonitrile containing formic acid (0.1%, *v/v*)		Eluent A: water containing difluoroacetic acid (0.1%, *v/v*) Eluent B: acetonitrile containing difluoroacetic acid (0.1%, *v/v*)

### Data processing and statistical analysis

Data processing for untargeted metabolomics was performed in a R environment according to previously published workflows (Hemmer et al., 2021; Manier et al., 2019a). Thermo Fisher LC-HRMS/MS RAW files were converted into mzXML files using ProteoWizard (Adusumilli & Mallick, 2017). XCMS parameters were optimized using a previously developed strategy as mentioned by Manier et al. (Manier et al., 2019a). Peak picking and alignment parameters are summarized in Table S2. Peak picking was performed using XCMS in an R environment (Smith et al., 2006; Team 2013) and the R package CAMERA (Kuhl et al., 2012) was used for the annotation of adducts, artifacts, and isotopes. Feature abundance with a value zero were replaced by the lowest measured abundance as a surrogate limit of detection and the whole dataset was then log 10 transformed (Wehrens et al., 2016). Normalization was performed for urine samples using the area of endogenous creatinine from those samples analyzed using HILIC column and positive ionization mode. For plasma samples, normalization was performed using the area of L-tryptophane-d_5_. The R scripts on GitHub (https://github.com/sehem/Columns_Metabolomics) and the mzXML files are available via Metabolights (study identifier MTBLS5082). The total feature count was used to evaluate the number of features detected by each analysis. Therefore, all adducts, artifacts, and isotopes annotated by CAMERA were removed (Kuhl et al., 2012). Subsequently, the QCs of each analysis were considered, since all features present in QCs should also be present in experimental groups. For the reproducibility of the features, the coefficient of variation (CV) was determined from the peak areas of the QCs. Significant changes of features between control and PCYP respectively blank and PCYP group were assumed after Welch’s two-sample *t*-test and Bonferroni correction for pHLM (Broadhurst & Kell, 2006), *p*-value < 0.01 for urine, and *p*-value < 0.05 for plasma. Principal component analysis (PCA) and hierarchal clustering were used to investigate patterns in the datasets. For pHLM, *t*-distributed stochastic neighborhood embedding (*t*-SNE) (van der Maaten, 2014; van der Maaten & Hinton, 2008) were used in addition to PCA. Names for features were adopted from XCMS using “M” followed by rounded mass and “T” followed by the retention time in seconds. After visual inspection of the extracted ion chromatograms (EIC) of significant features, based on the peak shape quality, the significant features were divided into true and false features (Hemmer et al., 2020).

## Results and discussion

**Fig. 1:**
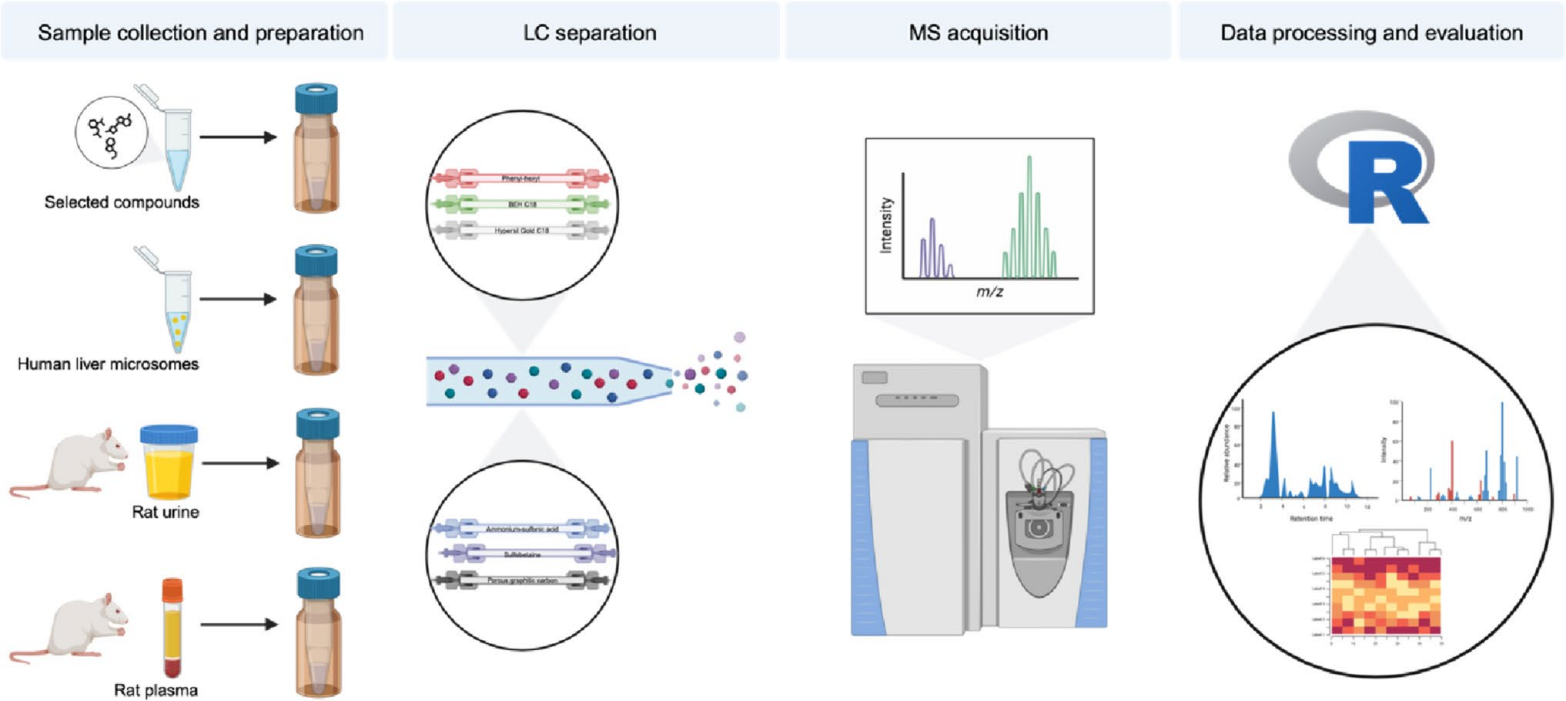
Overview of the analytical workflow used in this study. Sample types were prepared with different preparation methods; samples were then separated on different reversed-phase and hydrophilic interaction-phase columns; mass spectrometry acquisition was performed in positive and negative ionization mode; data processing and evaluation was done using an in-house R script based on XCMS; columns were compared in terms of their different outcomes. (Created with BioRender.com)

An overview of the workflow used in this study is given in Fig. 1. Since the aim of this study was to evaluate the influence of different analytical LC columns on the resolution of selected endogenous compounds and the results of untargeted metabolomics analyses, only columns were changed and other parameters remained unchanged. However, eluents and gradients had to be adopted and were selected according to the column types and as used in other studies (Hemmer et al., 2022; Manier et al., 2019b; Merck, 2019, 2020; Michely & Maurer, 2018). In addition, the choice of mobile phases was evaluated by the detectability of different compound classes using a system suitability test mixture described in the *Experimental Section* of the *Supplementary Information*. Sample preparations and all other LC-HRMS parameters such as column oven temperature, and MS settings were identical for all columns according to previously published studies (Hemmer et al., 2022; Manier et al., 2019b).

The Phenyl-Hexyl and Nucleodur columns were already used in previous studies and therefore used as reference for RP and HILIC analyses, respectively (Hemmer et al., 2020, 2021; Manier & Meyer, 2020; Manier et al., 2019b, 2020a, 2020b). However, since C_18_ columns are the most common RP columns (Harrieder et al., 2022), two differently linked C_18_ stationary phases were chosen over C_4_ or C_8_ phases. Criscuolo et al. showed that not all C_18_ columns are efficient for lipid separation and not only the chemistry of the stationary phase, but also the different types of particles or their sizes must be considered (Criscuolo et al., 2019). The Gold column, using spherical fully porous particles, was often used for screening and metabolomics methods (Imbert et al., 2021; Liu et al., 2021; Thevenot et al., 2015). The last of the selected RP columns, the BEH, consisted of a C_18_ modification with ethylene bridged hybrid (BEH) particles. It is expected to be a universal column with a wide pH range and was also used in other metabolomics studies previously (Gika et al., 2008; Tobin et al., 2021; Zhao et al., 2018).

Concerning HILIC, different stationary phase chemistries such as aminopropyl silane, alkyl amide, silica, or sulfobetaine groups are available. Amide or amino columns are one of the most frequently used HILIC columns. However, since these columns showed a reduced lifetime at elevated pH values, they were not included in this study (Harrieder et al., 2022). Instead, a sulfobetaine (ZicHILIC) column was selected, since it was often used in other metabolomics studies and showed suitable separation by its zwitterionic stationary phase (Abdalkader et al., 2021; Trivedi et al., 2012; Steuer et al., 2020). The in-house HILIC reference column, an ammonium-sulfonic acid (Nucleodur), has also a zwitterionic functional group.

According to the manufacturer, the porous graphitic carbon (PGC) column offers high column efficiency for polar compounds and improved retention of compounds normally only be retained with HILIC (Merck, 2019). PGC is also expected to show high robustness regarding the eluents, pH range, and pressure. Therefore, the PGC column was grouped together with the two HILIC columns but in contrast, PGC should demonstrate elevated stability with respect to pH value and allow retention of polar molecules without HILIC conditions (Bapiro et al., 2016; Hanai, 2003; Knox et al., 2006; Pereira, 2010). The performance of each column was tested before each run using the system suitability test mixture. In addition, the columns were equilibrated before each analysis as described in their care and use instructions.

Besides selected endogenous compounds such as amino acids, fatty acids, and sugars, three different datasets were generated and investigated by analyzing the following matrices. 1) pHLM incubations, a well-characterized in vitro model, which is commonly used in drug metabolism studies, since its ease of use and low variability (Asha & Vidyavathi, 2010; Richter et al., 2017). 2) Rat urine, a matrix to reflect the complexity of an in vivo model and which is rich in hydrophilic substances (Khamis et al., 2017; Wagmann et al., 2022). 3) Rat plasma, as a more complex matrix covering a broad spectrum of endogenous compound classes compared to urine.

### Resolution of selected endogenous compounds

Artificial mixtures of 34 compounds from classes such as amino acids, biogenic amines, carboxylic acids, fatty acids, sugars, and others, were investigated to conclude, which column might be most suitable for which compound class. The individual compounds and analytical results are shown in Table S1. The selection of compounds was made to ensure that relevant compound classes in endogenous metabolism were represented. The Phenyl-Hexyl and BEH columns exhibited quite similar behavior in terms of compound retention and retention time. In contrast, analytes eluted later by using the Gold column. With regard to mid- and non-polar substances, both arachidic acid and vitamin D_2_ were sufficiently retained using the Gold column in comparison to the Phenyl-Hexyl column. Regarding the HILIC columns, amino acids, carboxylic acids, and sugars could be sufficiently separated using both Nucleodur and ZicHILIC. Compared to the Nucleodur column, more amino acids and the carboxylic acids citrate and succinate were separated using the ZicHILIC column. With respect to biogenic amines, noradrenalin could not be retained by using any HILIC column. The PGC column was the only one of the six columns capable to retain the amino acid threonine. With respect to mid- and non-polar compounds, the PGC column was able to separate fewer substances than the two HILIC columns.

In summary, separation and retention of polar substances such as amino acids, carboxylic acids, biogenic amines, and sugars, ZicHILIC showed the best performance amongst all six columns, followed by Nucleodur. PGC was only able to separate and retain amino acids used in this study. Concerning the mid- and non-polar compounds, most of them were separated using the Gold column followed by the BEH and Phenyl-Hexyl columns. Compared to the Phenyl-Hexyl column, the used C_18_ columns are more suitable for separation of long-chain fatty acids since Phenyl-Hexyl columns are mainly designed to retain aromatic hydrocarbons. However, it is important to note that this is only a limited selection of compounds.

### Column performance

The performance of each column in terms of separation and chromatographic sensitivity (signal to noise ratio) was evaluated based on the peak-picking parameters obtained using QC samples (Table S2). Chromatographic peak width is important since narrow chromatographic peaks usually improve chromatographic sensitivity but in turn may reduce detection probability in slow mass analyzers. Broader peak shape usually leads to lower peak height (lower chromatographic sensitivity) and thus lower probability for being e.g., selected for fragmentation in data dependent approaches (Criscuolo et al., 2019). To evaluate the performance of each column, the minimum peak width was used to calculate peak capacity. Peak capacity is defined as the maximum number of peaks that can be chromatographically separated with a unit resolution within a retention time window using gradient elution and is directly proportional to the average peak resolution (Gilar et al., 2004; Wang et al., 2006). For this purpose, Eq. 1 was used to obtain the peak capacity *Pc* from the elution time *t*_*g*_ and the average peak width at baseline *W* (Neue, 2005).

1 $$Pc = 1 + \frac{t_g }{W}$$

Overall, the highest peak capacity for all three matrices was found after using PGC (Table S2). Between the two HILIC columns there is only slightly difference in peak capacity. In addition, there is no significant difference in peak capacity between the three RP columns.

The sensitivity of a system relates to the detector signal and the ability of peak to be chosen for MS/MS (Criscuolo et al., 2019). For evaluation of the sensitivity of each column, signal-to-noise threshold (snthresh) was used, which is defined as the ratio between the peak height from analytes to the peak height of background noise (Coleman et al., 2001). The highest snthresh ratio overall was shown using Gold and PGC column after analyzing rat urine and plasma (Table S2). For analyzing pHLM, Nucleodur showed the highest snthresh, whereas no differences were observed after using RP columns.

### Feature count

**Fig. 2:**
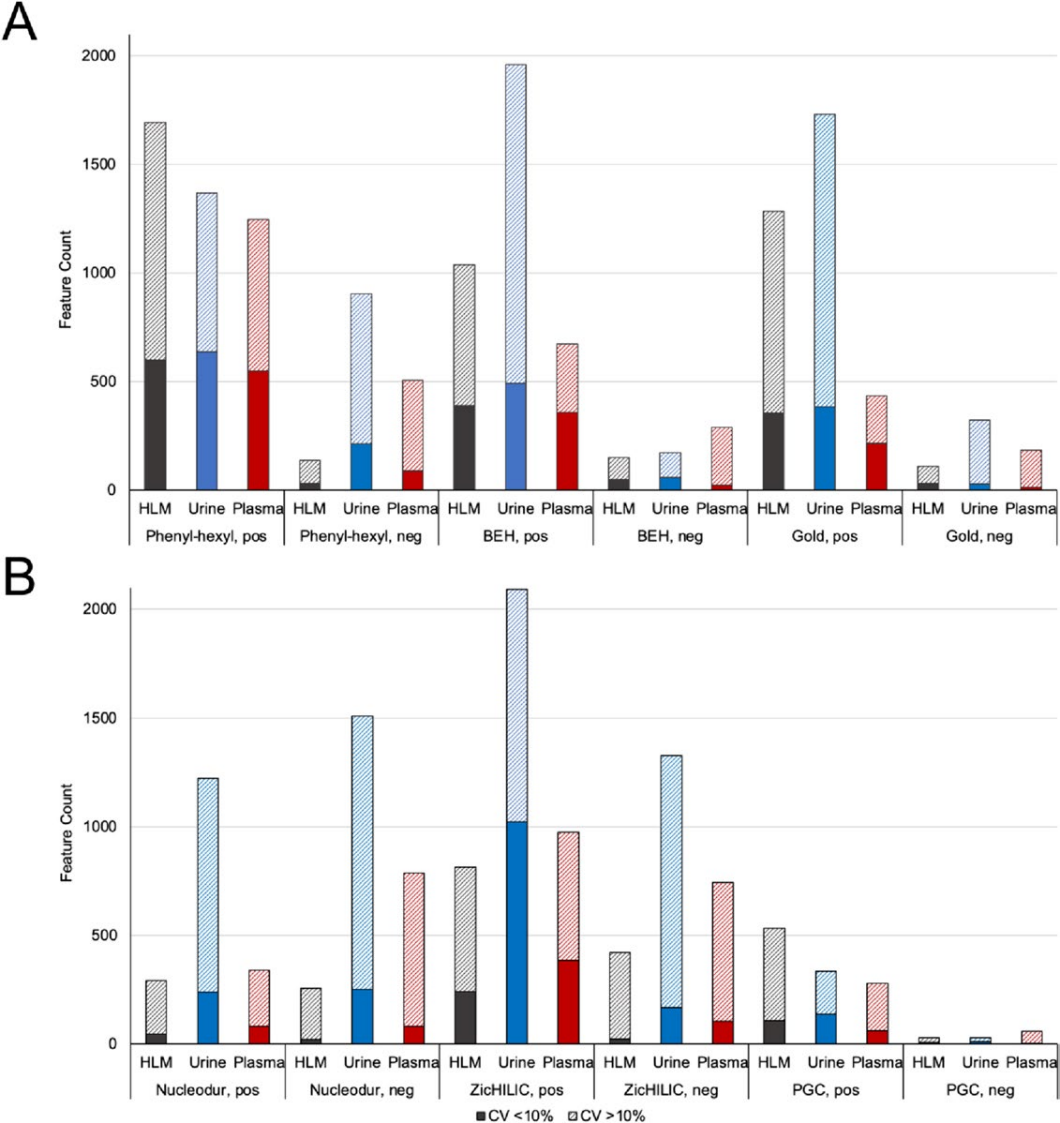
Bar chart showing feature count detected after peak picking and their respective reproducibility evaluated by CV (coefficient of variation) in pooled human liver microsomes (HLM), rat urine, and rat plasma using different reversed-phase (A) and HILIC (B) columns. pos = positive, neg = negative, BEH = BEH C_18_, Gold = Hypersil Gold C_18_, Nucleodur = ammonium-sulfonic acid, ZicHILIC = sulfobetaine, PGC = porous graphitic carbon

Features are chromatographic peaks detected by an algorithm and described by their retention time and their *m/z* (Mahieu et al., 2014). The size of the detected feature count is crucial for a sufficient description of e.g., the metabolome. Therefore, it can be assumed that the more features were detected after peak picking, the better the metabolome of the biosample was analytically described. However, it should be considered that the size of feature count can be influenced by non-matrix dependent parameters such as artifactual interference. These are peaks that originated from contaminants, chemical noise, and bioinformatic noise. In contrast, biologically derived features originated from metabolites of the analyzed biological sample. Therefore, a method that detects the maximum number of features is not always the method that provides the greatest metabolome coverage (Mahieu et al., 2014). In this study, the aim was to identify columns, that provide a sufficient metabolic coverage in term of number of feature count. In addition, the reproducibility of the features was also assessed by CV < 10%, to exclude possible artifactual interference. Figure 2 shows the feature count detected after peak picking (without isotopes and adducts detected by CAMERA) and their respective reproducibility evaluated by CV after analyzing all three matrices by using the six analytical columns and MS positive and negative ionization mode.

The feature count differed widely amongst the columns. The Phenyl-Hexyl and ZicHILIC columns allowed detection of most features across all three datasets. The urine metabolome, currently described by about 3,100 metabolites (Bouatra et al., 2013) seemed to be best covered after analysis using the BEH (1,960 features) and ZicHILIC columns (2,092 features) in positive mode. In contrast, the plasma metabolome was best described by the Phenyl-Hexyl and ZicHILIC columns. Since there are no data available on the number of metabolites in the plasma metabolome, the serum metabolome database was used as reference, which contains 4,651 small molecule metabolites (Psychogios et al., 2011). In comparison to the two other HILIC columns, significantly fewer features were detected in urine and plasma samples using the PGC. Reasons for this might be an inappropriate sample preparation, especially regarding the reconstitution solvent, or other LC parameters that were not further optimized in this study. Same patterns were observed for the reproducibility evaluated by CV < 10%. Again, both columns Phenyl-Hexyl and ZicHILIC show the highest number of reproducible features over all three matrices.

However, it should be considered that not all detected features are required to be of biological origin (Mahieu et al., 2014). Since the same samples of a dataset were always used for all six columns in this study, the number of artifactual features in the different analytical methods should be as low as possible and comparable. Contaminations originating from the samples themselves or from the sample processing can be excluded for the most part. However, differences in contamination may have occurred, for example due to the different eluents or stationary phases in the individual methods.

### Univariate and multivariate statistics

Univariate statistics aimed to identify those features that were significantly altered between control and experimental groups (Barnes et al., 2016b). They were done using Welch’s two-sample *t*-test. An overview of all detected significant features can be found in Table S3 (sheet 1–3) in the *Supplementary Information*. No significant features were found after analyzing pHLM using MS in negative ionization mode independent from the used column. In addition, no significant features were found after analyzing rat urine by PGC and MS in negative mode as well as after analyzing rat plasma by the Gold column and MS in negative mode as well as PGC and MS in positive and negative mode.

Nevertheless, the columns were also evaluated according to the peak shape quality of the significant features. Since the EIC of some significant features turned out to be false features, they were divided into true and false features based on the peak shape quality of their EIC according to the criteria used by Hemmer et al. (Hemmer et al., 2020). Therefore, the ratio of false vs true features was calculated (Table S4). Over all three datasets, the Phenyl-Hexyl column and the ZicHILIC showed the lowest ratio followed by Gold and Nucleodur columns.

Besides univariate statistics, the different columns were also evaluated regarding the results of multivariate statistics to identify the largest changing features and specific signatures in the data. Since multivariate statistics can only be performed if there were at least two significant features, no data were available for datasets containing no or only one significant feature. In this study, PCA and hierarchical clustering were used to discover differences between the columns. The figures for the different datasets can be found in the *Supporting Information* (Figure S1–14). It was shown that groups blank vs PCYP and control vs PCYP, were distinct from each other independent from the used column and investigated matrix. Since the results of the variance of the first principal component indicated that the pHLM datasets were highly linear (Figure S1, 2), the patterns in pHLM dataset were evaluated using *t*-SNE (van der Maaten & Hinton, 2008). Results of the *t*-SNEs (Figure S7, 8) showed similar cluster patterns for all columns. Regarding hierarchical clustering, there was in general a high distance between samples from blank or control group to those from PCYP and QC group (Figure S9–14), again independent from the used columns and investigated matrix. Therefore, it can be assumed that with respect to the multivariate statistics, there should be no significant influence of the used column on its outcome. After separation using PGC, no significant features were found in the plasma data, and thus, no multivariate statistics were performed. One explanation for this might be the different compositions of plasma and urine. While lipids and similar compounds predominate in plasma, more polar substances are present in urine (Bouatra et al., 2013; Psychogios et al., 2011). The PGC column should be much better suited for polar substances, such as those found in urine.

## Summary of column comparison

Table 2 provides a brief summary of the results described above for each column. With respect to the different matrices, the Phenyl-Hexyl column was well suited for all three matrices, concerning both the overall number of features and the reproducibility of them. In addition, the Phenyl-Hexyl column exhibits a low false feature rate compared to C_18_ columns. Compared to the Phenyl-Hexyl column, the two C_18_ columns performed similarly. BEH showed significantly more features in urine compared to both other columns.

**Table 2:** Summary of study factors and the respective results from this study

Parameter	Reversed-phase columns			Hydrophilic interaction columns		
	Phenyl-Hexyl	BEH	Gold	Nucleodur	ZicHILIC	PGC
High chromatographic resolution	Short FA, Steroids	Long FA	FA, steroids	AA, BA, sugars	AA, BA, CA, sugars	AA
Column performance (peak width, peak capacity, snthresh)	High	Low	High	Low	Low	High
Feature count	High	Low	Low	Low	High	Low
Reproducibility feature count	High	Low	Low	Low	High	Low
False feature rate	Low	High	Low	High	Low	Low
Recommended matrix	H, U, P	H, U	H, U	P	H, U, P	H

Each column is compared within their chromatographic technique. BEH = BEH C_18_, Gold = Hypersil Gold C_18_, Nucleodur = ammonium-sulfonic acid, ZicHILIC = sulfobetaine, PGC = porous graphitic carbon, AA = amino acids, CA = carboxylic acids, BA = biogenic amines, FA = Fatty acids, H = pooled human liver microsomes, U = rat urine, P = rat plasma

Regarding the different matrices, the ZicHILIC column showed the best performance for analysis of urine and pHLM represented by e.g., the lowest false feature rate. Compared to the other two HILIC columns, the lowest number of features were detected after PGC separation. Despite initial optimization using the system suitability test mixture, further optimization seems to be needed for the PCG column. However, this should be aim of another study. Another explanation might be the composition of the metabolites in the different matrices. Compared to plasma, urine contains more polar compounds, which can be better separated by PGC (Bouatra et al., 2013; Psychogios et al., 2011). Nevertheless, PGC showed better peak capacity and snthresh than the HILIC columns. In terms of compound classes, the PGC column performed well for separation of amino acids. The ZicHILIC and Nucleodur column were equally suitable for the separation of polar substances such as amino acids or sugars.

In summary, even though the chemistry of the stationary phase remains the same, there are significant differences between the investigated columns. Results of this study confirmed that the LC columns should be adapted to both the matrix and metabolites being investigated.

### Limitations of the study

The present study provides only a small insight into how different analytical columns can affect the outcome of an untargeted metabolomics study. The study also used only a limited selection from a huge pool of columns and the dimensions of the different columns were not identical. It is known that the column geometry and the particle size can play a crucial role (Criscuolo et al., 2019).

As preliminary experiments had shown that not every eluent was suitable for all columns, eluents could not be kept consistent and had to be slightly adapted. Since the study was primarily based on an untargeted approach, selected endogenous compounds were still used to detect any differences between the columns with respect to different compound classes. The results of the study indicate that selecting the most appropriate column for both the investigated matrix and compounds is crucial for interpretation of an untargeted metabolomics study. For example, if the researcher wants to detect as many metabolites as possible, the Phenyl-Hexyl column may provide the best results for all three matrices. However, certain classes of compounds, such as long fatty acids, may be less detectable with the Phenyl-Hexyl column. If the researcher wants to keep the analytical setup the same for all investigated matrices, the Phenyl-Hexyl column might be suitable. However, if the researcher chooses the more time-consuming and costly route, different analytical methods should be used for each matrix. For example, BEH for rat urine samples.

## Conclusion

Using LC-(MS), the choice of the best suitable analytical columns plays a crucial role since the metabolome includes many compound classes with high chemical diversity and complexity. Thus, the influence of different reversed-phase, HILIC, and PGC columns was investigated on the outcome of an untargeted metabolomic study using three different matrices. Evaluation criteria included e.g., peak capacity, size of feature count, and results of multivariate statistics.

The study showed that a combination of BEH and ZicHILIC might be a suitable choice for analysis of urine samples and a combination of Phenyl-Hexyl and ZicHILIC might be suitable for analysis of plasma samples. Over all three datasets, the best results were obtained by using a combination of Phenyl-Hexyl and ZicHILIC. However, concerning the use of Phenyl-Hexyl column for reversed-phase, it should be considered that mainly non-polar metabolites with aromatic hydrocarbon structure can be retained, and that e.g., fatty acids may not retain. Considering the results of this study, it can be concluded that if researchers want to achieve the best possible results, they should test and adapt the analytical method for each matrix and set of investigated substances.

### Supplementary Information

Below is the link to the electronic supplementary material.

Supplementary file1 (XLSX 24 KB)

Supplementary file2 (DOCX 2235 KB)

## Data Availability

The R script can be found on GitHub (https://github.com/sehem/Columns_Metabolomics) and the mzXML files used in this study are available via Metabolights (www.ebi.ac.uk/metabolights/MTBLS5082).
