# Supplementary material for: Comparison of reversed-phase, hydrophilic interaction, and porous graphitic carbon chromatography columns for an untargeted toxicometabolomics study in pooled human liver microsomes, rat urine, and rat plasma
Source: Metabolomics. 2024 Apr 30;20(3):49. doi: 10.1007/s11306-024-02115-0 (PMC11061011; doi:10.1007/s11306-024-02115-0)
Supplement: Supplementary file 2 — Supplementary file2 (DOCX 2235 KB) [file 11306_2024_2115_MOESM2_ESM.docx]

**Supporting Information**

**Comparison of reversed-phase, hydrophilic interaction, and porous graphitic carbon chromatography columns for untargeted toxicometabolomics study of pooled human liver microsomes, rat urine, and rat plasma**

Selina Hemmer, Sascha K. Manier, Lea Wagmann, Markus R. Meyer

# Experimental section

## Performance test of each column using a system suitability test mixture

The performance of each column was tested before each measurement. For this purpose, a test mixture was used, which contained the following analytes: Glucose-d_7_ (10 mg/L), creatinine-d_3_ (1 mg/L), tryptophane-d_5_ (10 mg/L), cortisol (10 mg/L), pregnenolone (10 mg/L), prostaglandin-E3-d_9_ (10 mg/L), 1-palmitoyl-d_9_-2-palmitoyl-*sn*-glycero-3-PC (10 mg/L), and palmitic acid-d_31_ (20 mg/L). The analytes were spiked in methanol for the phenyl-hexyl column. For the Hypersil Gold C_18_ (Gold), BEH C_18_ (BEH), ammonium-sulfonic acid (Nucleodur), and sulfobetaine (ZicHILIC) columns, analytes were spiked in acetonitrile, and for porous graphitic carbon (PGC) column analytes were spiked in water containing DFA (0.1 %, *v/v*).

## Identification of significant features

Significant features were identified by recording MS/MS spectra using the PRM method mentioned above. After conversion to mzXML format using ProteoWizard (Adusumilli and Mallick, 2017), spectra were imported to NIST MS Search version 2.3 Library and the settings for library, and MS/MS search were used according to published procedures (Hemmer *et al.*, 2020; Hemmer *et al.*, 2021; Hemmer *et al.*, 2022; Manier *et al.*, 2020). Metabolites of the synthetic cathinone PCYP were tentatively identified by interpreting their spectra in comparison to that of the parent compound. Identified features were classified on the different levels of identification according to the Metabolomics Standards Initiative (MSI) (Sumner *et al.*, 2007).

**Table S1.** Overview of the detected compound classes using different reversed-phase and hydrophilic interaction chromatography columns sorted by compound class. Retention times of the compounds detected utilizing the respective columns are given in seconds (s). BEH = BEH C_18_, Gold = Hypersil Gold C_18_, Nucleodur = ammonium-sulfonic acid, ZicHILIC = sulfobetaine, PGC = porous graphitic carbon. Hyphen (-) means that neither a peak nor a MS^2^ were detected for this compound using the corresponding column.

| Compound class | Compound | RT reversed-phase, s | | | RT HILIC, sec | | |
| --- | --- | --- | --- | --- | --- | --- | --- |
|  |  | Phenyl-hexyl | BEH | Gold | Nucleodur | ZicHILIC | PGC |
| Amino acid | Creatinine | 26 | 29 | 59 | 356 | 293 | 191 |
| Amino acid | Glutamine | 26 | 27 | 57 | 511 | 466 | 73 |
| Amino acid | Glutaminic acid | 26 | 27 | 57 | 533 | 488 | 81 |
| Amino acid | Histidine | 23 | 27 | 55 | 552 | 484 | 103 |
| Amino acid | Kynurenine | 88 | 127 | 188 | 446 | 364 | 296 |
| Amino acid | Lysin | 23 | 27 | 55 | 550 | 518 | 47 |
| Amino acid | Proline | 28 | 29 | 59 | 490 | 436 | 61 |
| Amino acid | Threonine | - | - | - | - | - | 53 |
| Amino acid | Tryptophane | 149 | 172 | 217 | 452 | 380 | 344 |
| Biogenic amine | Carnosine | 24 | 28 | 52 | 550 | 506 | 190 |
| Biogenic amine | Dopamine | 38 | 57 | 95 | 443 | 400 | 239 |
| Biogenic amine | Histamine | 22 | 28 | 50 | 500 | 440 | 89 |
| Biogenic amine | Noradrenalin | 26 | 32 | 59 | - | - | - |
| Biogenic amine | Serotonin | 71 | 126 | 179 | 434 | 381 | - |
| Biogenic amine | Spermidine | 22 | 37 | 50 | 620 | 649 | - |
| Carboxylic acid | Citrate | 35 | 33 | 76 | 538 | 503 | - |
| Carboxylic acid | Succinate | 52 | 61 | 109 | - | 456 | - |
| Coenzyme | NAD | 32 | 52 | 80 | - | - | - |
| Fatty acid | Arachidic acid | - | 697 | 674 | 82 | 44 | - |
| Fatty acid | Lauric acid | 421 | - | - | - | - | - |
| Lipide | Cholesteryl oleate | - | - | - | - | - | - |
| Nucleotide | Adenosine 5’ Diphosphate | - | - | - | - | - | - |
| Nucleotide | Guanosine 5’ Triphosphate | - | - | - | - | - | - |
| Nucleotide | Inosine | 61 | 98 | 151 | 367 | 329 | 349 |
| Steroid | 25-HO Cholesterol | - | - | - | - | - | - |
| Steroid | Cortisone | 331 | 310 | 323 | 92 | 52 | - |
| Steroid | Vitamin D2 | - | - | 699 | - | - | - |
| Sugar | D-Fructose | - | - | - | 490 | 707 | - |
| Sugar | D-Glucose | 25 | 28 | 43 | 481 | 445 | - |
| Sugar | D-Ribose | 26 | 29 | 44 | 503 | 296 | - |
| Sugar | Maltose | 26 | 29 | 41 | 257 | 463 |  |
| Vitamin | Ascorbate | - | - | - | 536 | 713 | - |
| Vitamin | Riboflavin | 211 | 214 | 245 | 329 | 263 | - |
| Vitamin | Retinol | - | - | - | - | - | - |

**Table S2.** Overview of peak picking and alignment parameters used for preprocessing and calculation of peak capacity for different columns and respective matrices. BEH = BEH C_18_, Gold = Hypersil Gold C_18_, Nucleodur = ammonium-sulfonic acid, ZicHILIC = sulfobetaine, PGC = porous graphitic carbon, pos = positive, neg = negative, ppm = allowed ppm deviation of mass traces for peak picking, snthresh = signal-to-noise threshold, mzdiff = minimum difference in m/z for two peaks to be considered as separate, prefilter 1 = minimum of scan points, prefilter 2 = minimum abundance, bw = bandwidth for grouping of peaks across separate chromatograms.

| Column | Matrix | Polarity | Peak width, s | Peak width, max | ppm | sntresh | mzdiff | Prefilter 1 | Prefilter 2 | bw | Peak capacity |
| --- | --- | --- | --- | --- | --- | --- | --- | --- | --- | --- | --- |
| Phenyl-hexyl | pHLM | pos | 8.9 | 100 | 1.8 | 10 | 0.018 | 7 | 100 | 5 | 92 |
|  |  | neg | 8.9 | 15 | 1.7 | 27 | 0.094 | 5 | 100 | 1 | 92 |
|  | Urine | pos | 8.9 | 19 | 1 | 12 | 0.012 | 7 | 100 | 2.5 | 92 |
|  |  | neg | 7.8 | 15 | 2.5 | 18 | -0.098 | 6 | 100 | 4.5 | 105 |
|  | Plasma | pos | 8.9 | 33 | 1.3 | 12 | 0.1 | 7 | 100 | 1 | 92 |
|  |  | neg | 6.8 | 100 | 1.8 | 16 | 0.01 | 5 | 100 | 1 | 102 |
| BEH | pHLM | pos | 8.9 | 12 | 1.6 | 13 | 0.016 | 5 | 100 | 1.5 | 92 |
|  |  | neg | 8.9 | 33 | 1.4 | 15 | 0.1 | 7 | 100 | 1 | 92 |
|  | Urine | pos | 7.8 | 21 | 2.4 | 12 | -0.098 | 6 | 100 | 1 | 105 |
|  |  | neg | 8.9 | 10 | 1.4 | 22 | 0.002 | 8 | 100 | 1.5 | 92 |
|  | Plasma | pos | 9.9 | 12 | 1.5 | 14 | 0.096 | 6 | 100 | 0.2 | 83 |
|  |  | neg | 8.9 | 93 | 2.5 | 13 | -0.002 | 5 | 100 | 0.3 | 92 |
| Gold | pHLM | pos | 8.9 | 15 | 2.5 | 12 | 0.1 | 6 | 100 | 2 | 92 |
|  |  | neg | 8.9 | 15 | 1.2 | 30 | -0.002 | 7 | 100 | 1 | 92 |
|  | Urine | pos | 8.9 | 17 | 2.5 | 12 | 0.004 | 5 | 100 | 1 | 92 |
|  |  | neg | 7.8 | 100 | 1.4 | 23 | -0.1 | 1 | 2100 | 0.8 | 105 |
|  | Plasma | pos | 8.9 | 15 | 2.5 | 54 | 0.1 | 6 | 100 | 0.2 | 92 |
|  |  | neg | 7.8 | 100 | 2.5 | 45 | 0.1 | 5 | 100 | 1 | 105 |

**Table S2.** Continued.

| Column | Matrix | Polarity | Peak width, s | Peak width, max | ppm | sntresh | mzdiff | Prefilter 1 | Prefilter 2 | bw | Peak capacity |
| --- | --- | --- | --- | --- | --- | --- | --- | --- | --- | --- | --- |
| Nucleodur | pHLM | pos | 9.9 | 100 | 2.5 | 42 | 0.1 | 5 | 100 | 1.5 | 74 |
|  |  | neg | 7.8 | 15 | 2.1 | 56 | 0.1 | 7 | 8000 | 1 | 93 |
|  | Urine | pos | 9 | 20 | 2.5 | 14 | 0.0059 | 7 | 100 | 1.5 | 81 |
|  |  | neg | 8.9 | 37 | 2.5 | 18 | 0.038 | 7 | 100 | 1 | 82 |
|  | Plasma | pos | 7.8 | 91 | 1.1 | 13 | 0.014 | 1 | 100 | 1 | 93 |
|  |  | neg | 8.9 | 33 | 2.5 | 11 | 0.014 | 1 | 100 | 0.9 | 82 |
| ZicHILIC | pHLM | pos | 7.8 | 29 | 1.6 | 17 | 0.006 | 6 | 100 | 0.5 | 93 |
|  |  | neg | 7.8 | 17 | 2.5 | 51 | 0.01 | 6 | 1300 | 1 | 93 |
|  | Urine | pos | 8.9 | 21 | 1.9 | 16 | 0.02 | 8 | 100 | 1.5 | 82 |
|  |  | neg | 8.9 | 35 | 1.3 | 15 | 0.022 | 8 | 100 | 1.5 | 82 |
|  | Plasma | pos | 8.9 | 46 | 1.4 | 6 | 0.034 | 6 | 100 | 0.2 | 82 |
|  |  | neg | 8.9 | 25 | 2.5 | 15 | 0.034 | 6 | 100 | 0.9 | 82 |
| PGC | pHLM | pos | 7.8 | 15 | 1.2 | 12 | 0.002 | 6 | 100 | 1 | 105 |
|  |  | neg | 8.9 | 15 | 1.4 | 25 | 0.1 | 16 | 100 | 0.5 | 92 |
|  | Urine | pos | 9 | 15 | 1.5 | 30 | 0.024 | 5 | 100 | 1 | 91 |
|  |  | neg | 7.9 | 12 | 1.8 | 14 | 0.002 | 36 | 100 | 0.5 | 104 |
|  | Plasma | pos | 8.9 | 12 | 1.2 | 42 | 0.066 | 6 | 100 | 0.9 | 92 |
|  |  | neg | 5.8 | 44 | 2 | 4 | 0.1 | 54 | 100 | 1 | 141 |

**Table S3.** Overview of the significant features using different columns in the corresponding matrices, namely pooled human liver microsome (pHLM) incubation (sheet 1), rat urine (sheet 2), and rat plasma (sheet 3) in which the features showed significant changes between PCYP and blank or control group. Features are sorted according to m/z values, followed by the polarity, the retention time (RT) for the corresponding column in seconds (s), identity, and the identification level according to MSI. BEH = BEH C_18_, Gold = Hypersil Gold C_18_, Nucleodur = ammonium-sulfonic acid, ZicHILIC = sulfobetaine, PGC = porous graphitic carbon. Hyphen (-) means that the feature was not significant using the corresponding column.

**Table S4.** Overview of the calculated false-positive rates of the significant features for different columns and the respective matrices. Pos = positive, neg = negative, BEH = BEH C_18_, Gold = Hypersil Gold C_18_, Nucleodur = ammonium-sulfonic acid, ZicHILIC = sulfobetaine, PGC = porous graphitic carbon

|  | Phenyl-hexyl pos | Phenyl-hexyl neg | BEH pos | BEH neg | Gold pos | Gold neg |
| --- | --- | --- | --- | --- | --- | --- |
| pHLM | 0 % | - | 4 % | - | 0 % | - |
| Urine | 10 % | 59 % | 53 % | 0 % | 55 % | 57 % |
| Plasma | 13 % | 100 % | 50 % | 0 % | 0 % | - |
|  | Nucleodur pos | Nucleodur neg | ZicHILIC pos | ZicHILIC neg | PGC pos | PGC neg |
| pHLM | 0 % | 50 % | 8 % | - | 0 % | - |
| Urine | 25 % | 55 % | 29 % | 35 % | 0 % | - |
| Plasma | 15 % | 63 % | 17 % | 40 % | - | - |


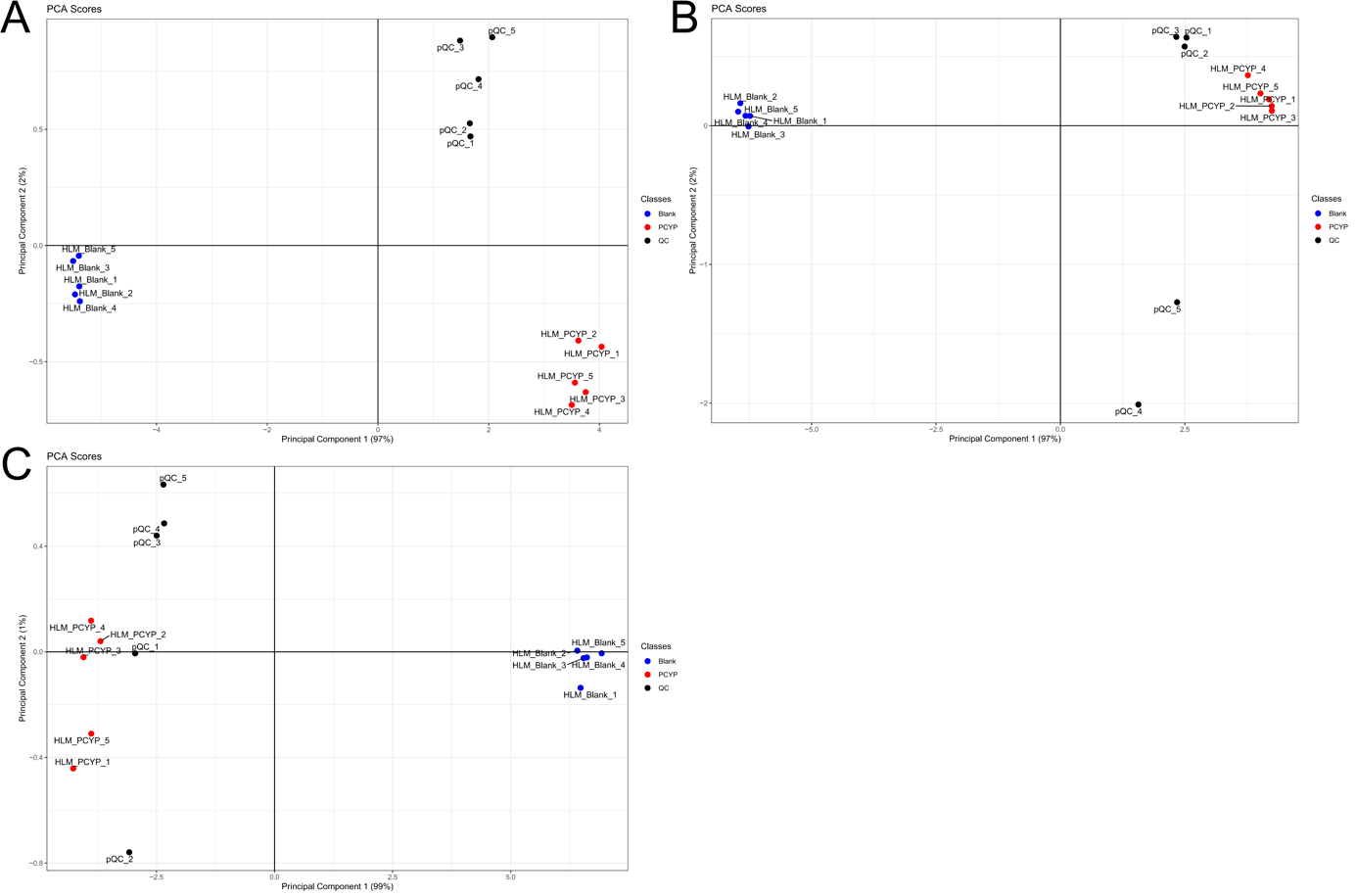


**Figure S1.** Scores of principal component analysis of pooled human liver microsome samples after analysis using reversed-phase chromatography in positive ionization mode. A = Phenyl-hexyl, B = BEH, and C = Gold.


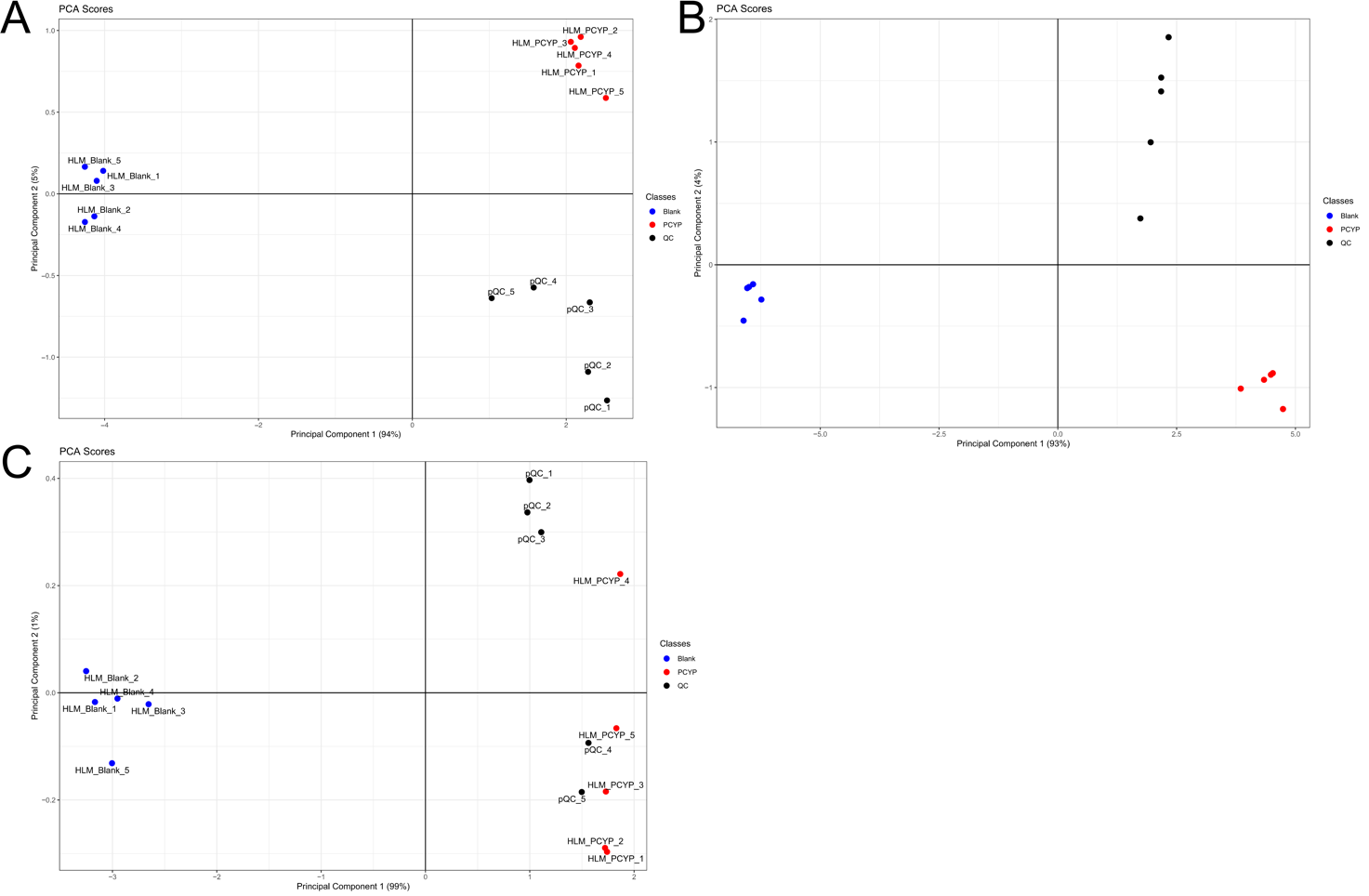


**Figure S2.** Scores of principal component analysis of pooled human liver microsome samples after analysis using hydrophilic interaction chromatography in positive ionization mode. A = Nucleodur, B = ZicHILIC, and C = PGC.


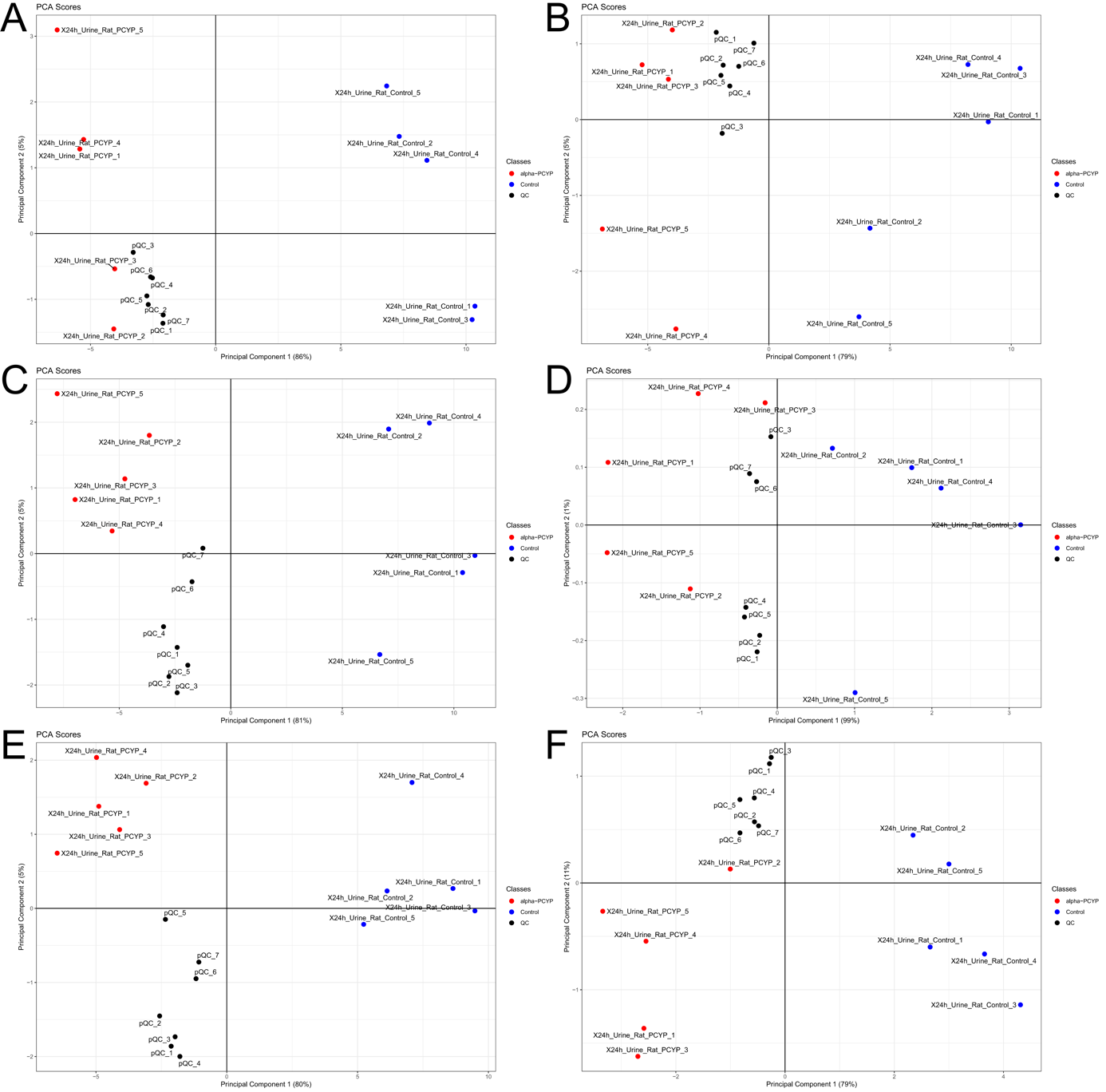


**Figure S3.** Scores of principal component analysis of rat urine samples after analysis using reversed-phase chromatography in positive (pos) and negative (neg) ionization mode. A = Phenyl-hexyl pos, B = Phenyl-hexyl neg, C = BEH pos, D = BEH neg, D = Gold pos, and F = Gold neg.


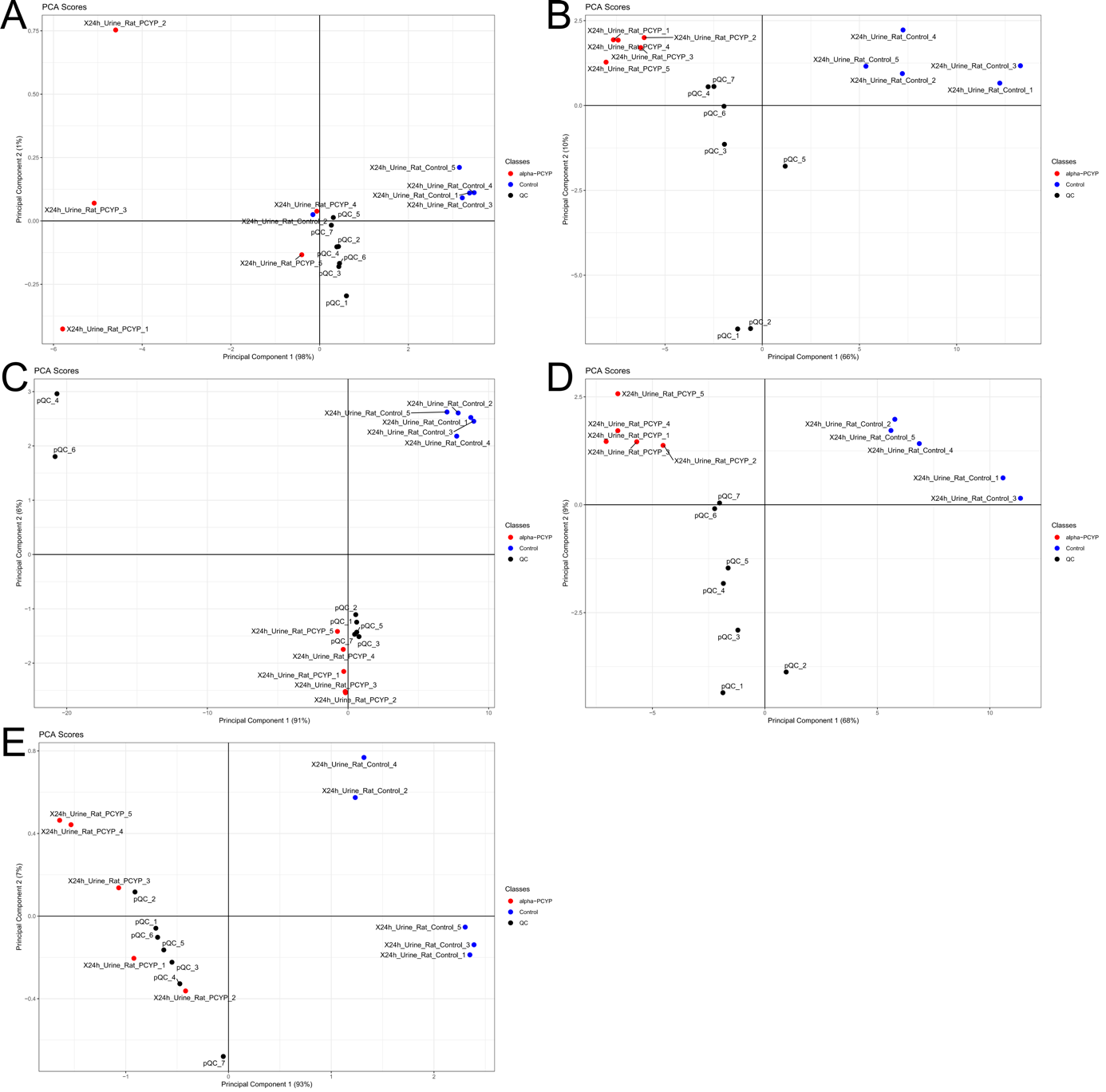


**Figure S4.** Scores of principal component analysis of rat urine samples after analysis using hydrophilic interaction chromatography in positive (pos) and negative (neg) ionization mode. A = Nucleodur pos, B = Nucleodur neg, C = ZicHILIC pos, D = ZicHILIC neg, and E = PGC pos.


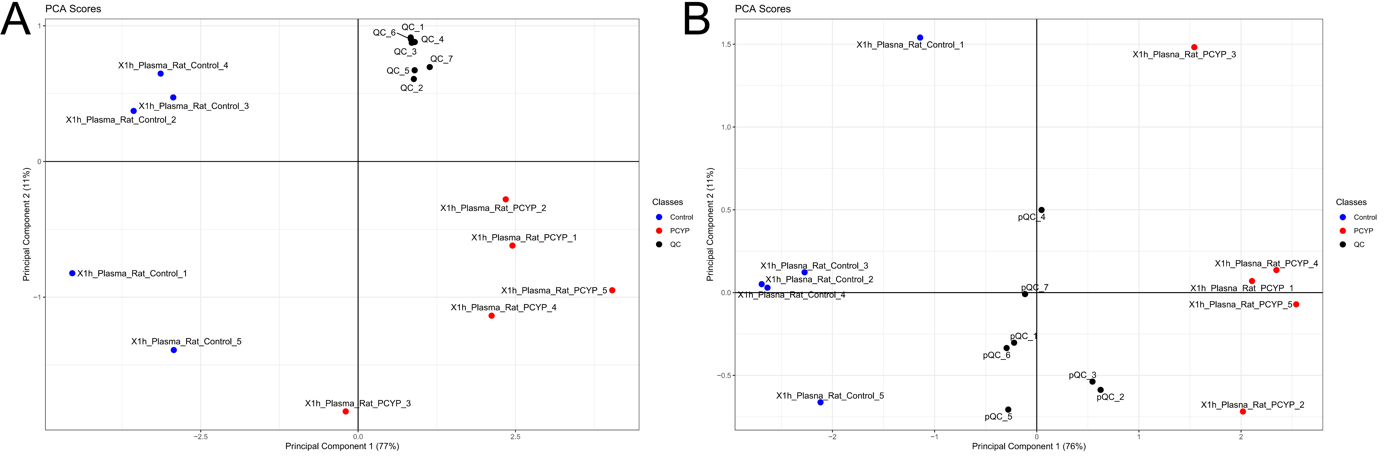


**Figure S5.** Scores of principal component analysis of rat plasma samples after analysis using reversed-phase chromatography in positive ionization mode. A = Phenyl-hexyl and B = BEH.


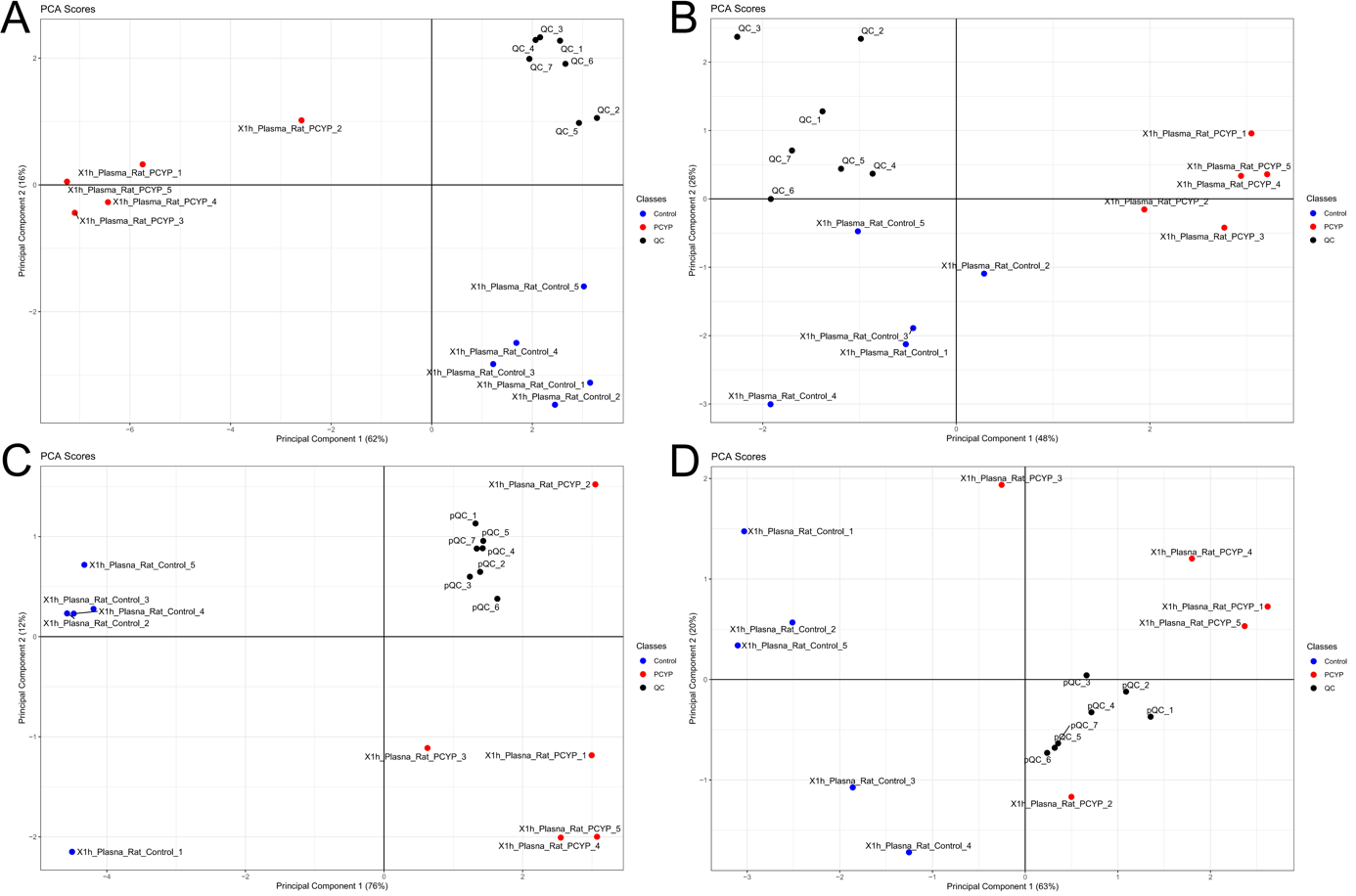


**Figure S6.** Scores of principal component analysis of rat plasma samples after analysis using hydrophilic interaction chromatography in positive (pos) and negative (neg) ionization mode. A = Nucleodur pos, B = Nucleodur neg, C = ZicHILIC pos, and D = ZicHILIC neg.


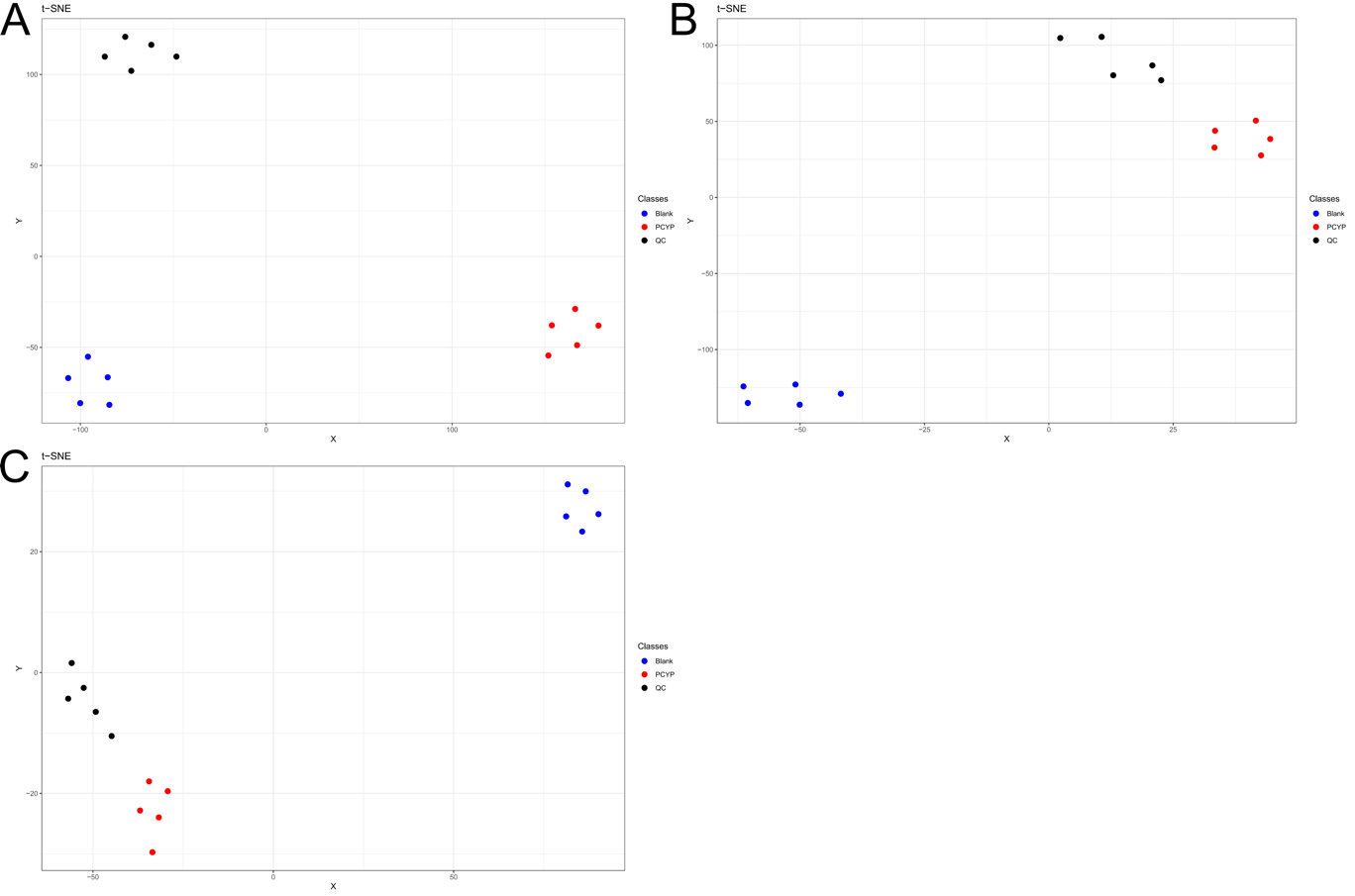


**Figure S7.** Results of t-distributed stochastic neighborhood embedding (t-SNE) of pooled human liver microsome samples after analysis using reversed-phase chromatography in positive ionization mode. A = Phenyl-hexyl, B = BEH, and C = Gold.


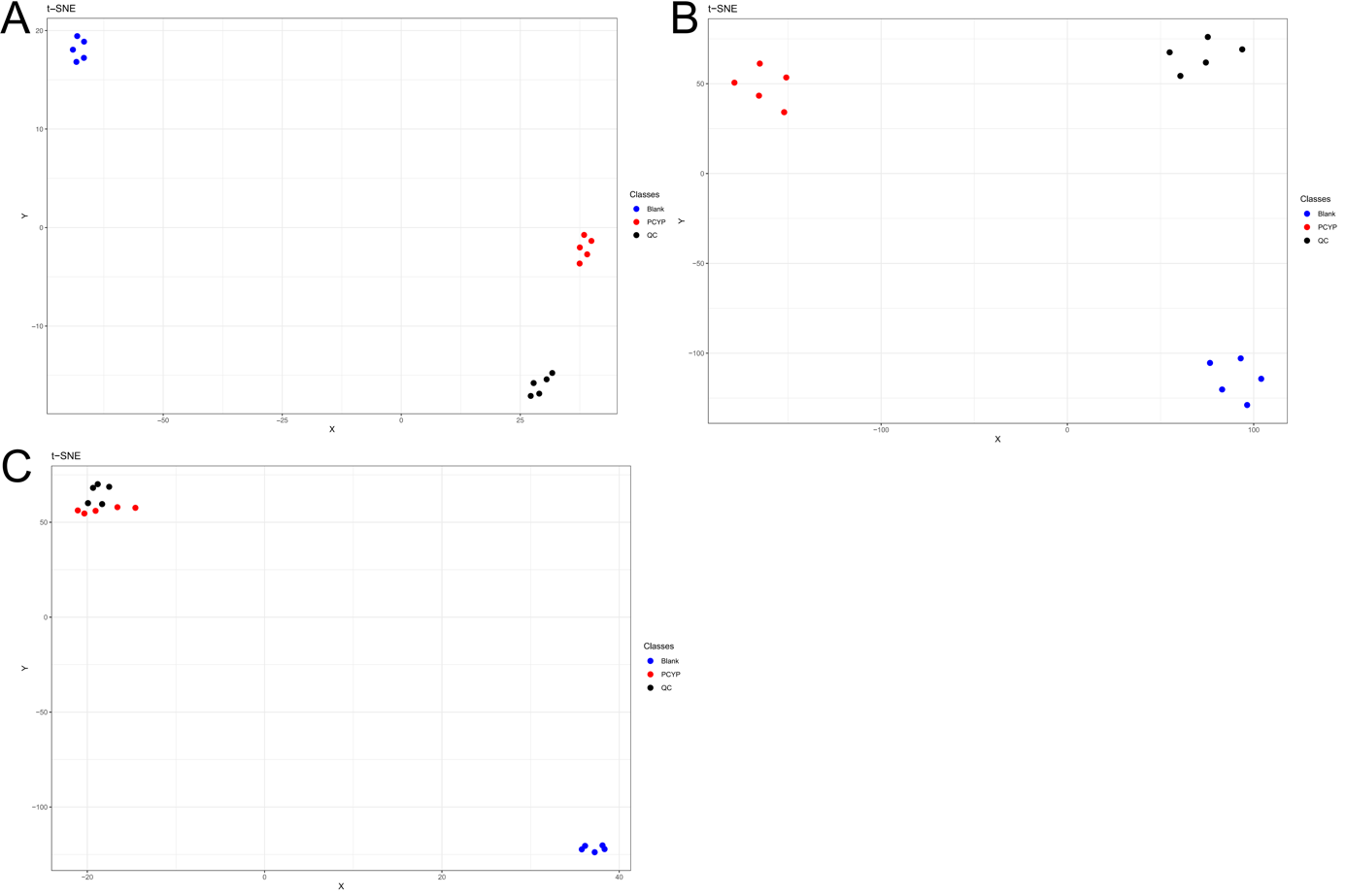


**Figure S8.** Results of t-distributed stochastic neighborhood embedding (t-SNE) of pooled human liver microsome samples after analysis using hydrophilic interaction chromatography in positive ionization mode. A = Nucleodur, B = ZicHILIC, and C = PGC.


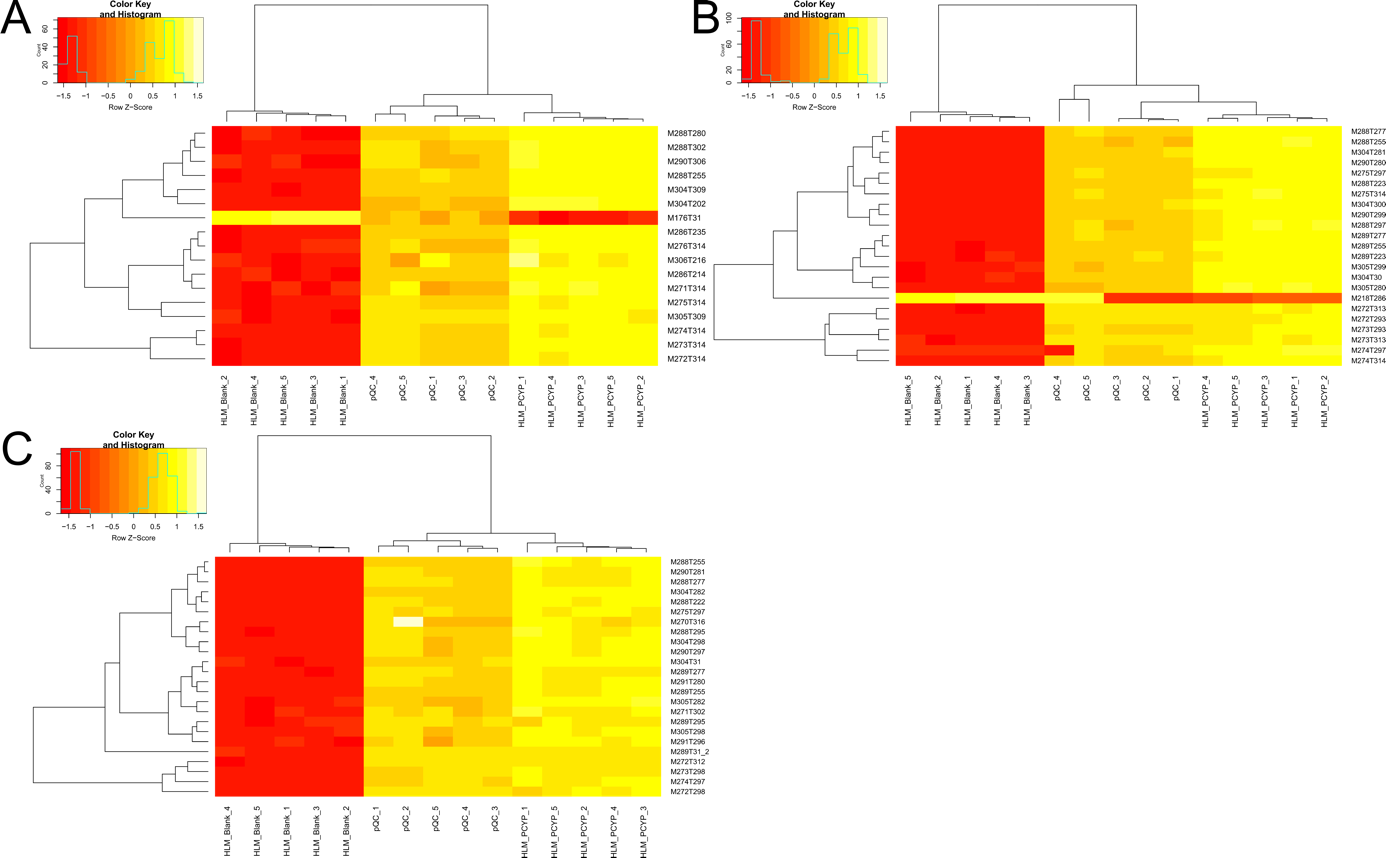


**Figure S9.** Results of heat map of hierarchical clustering of pooled human liver microsome samples after analysis using reversed-phase chromatography in positive ionization mode. A = Phenyl-hexyl, B = BEH, and C = Gold.


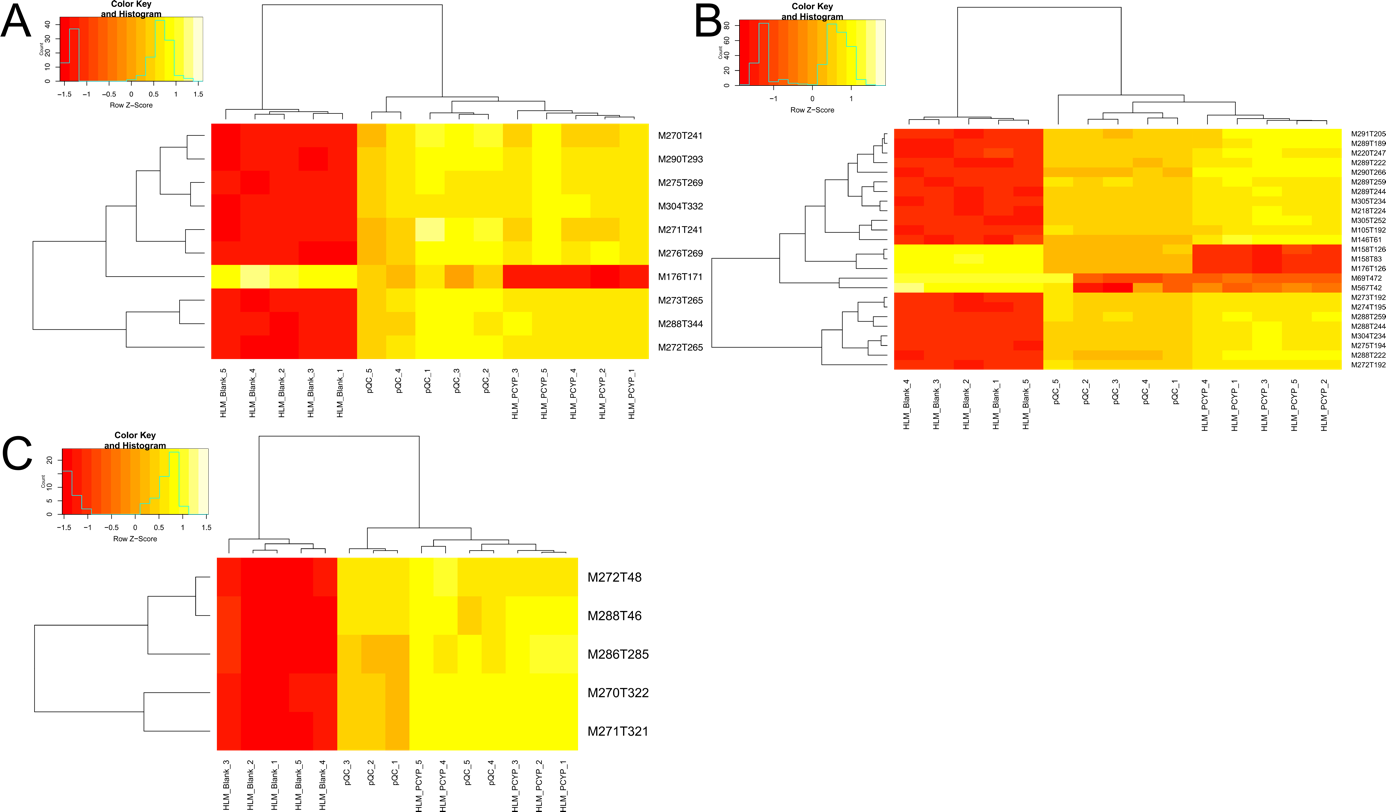


**Figure S10.** Results of heat map of hierarchical clustering of pooled human liver microsome samples after analysis using hydrophilic interaction chromatography in positive ionization mode. A = Nucleodur, B = ZicHILIC, and C = PGC.


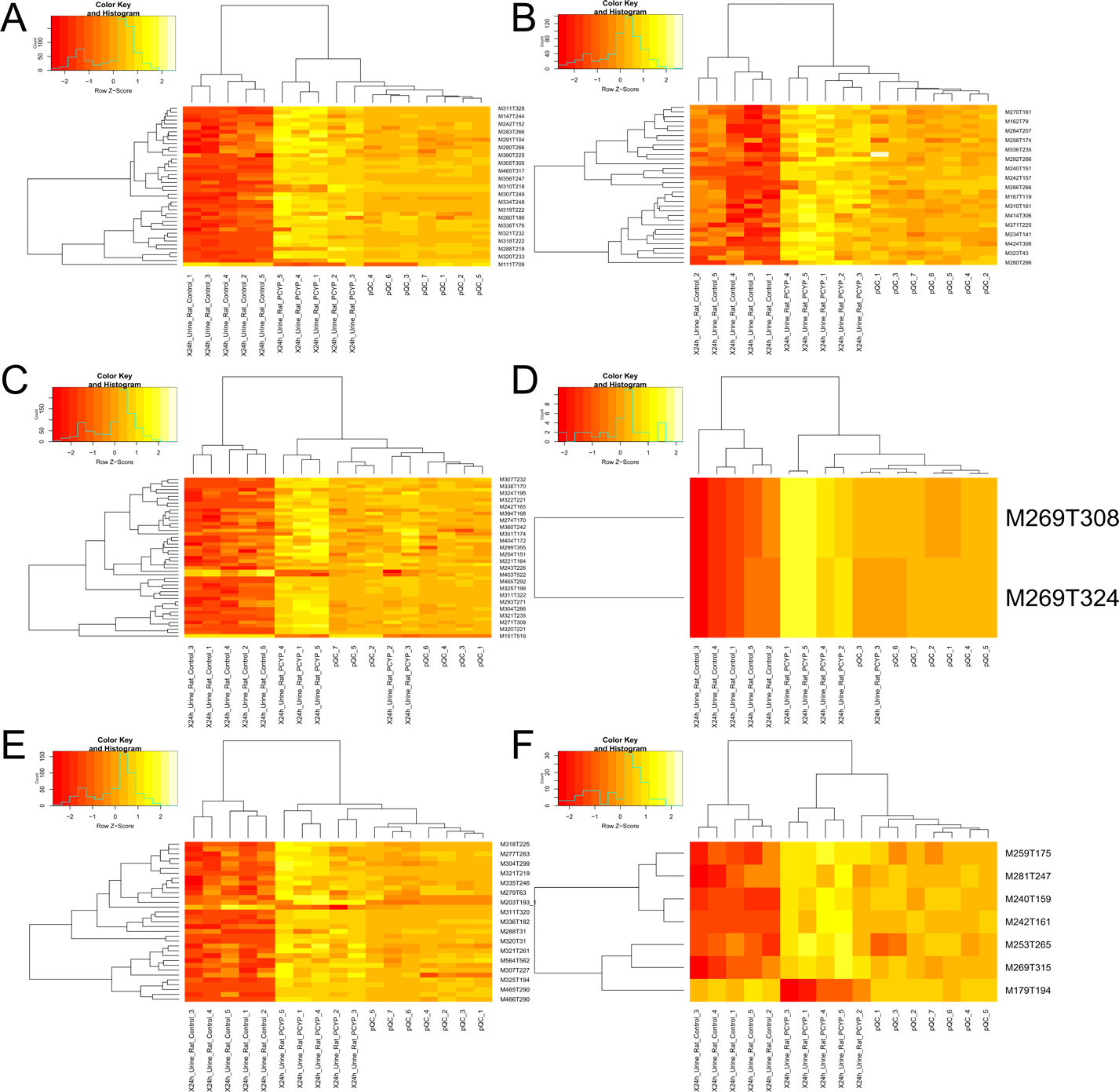


**Figure S11.** Results of heat map of hierarchical clustering of rat urine samples after analysis using reversed-phase chromatography in positive (pos) and negative (neg) ionization mode. A = Phenyl-hexyl pos, B = Phenyl-hexyl neg, C = BEH pos, D = BEH neg, D = Gold pos, and F = Gold neg.


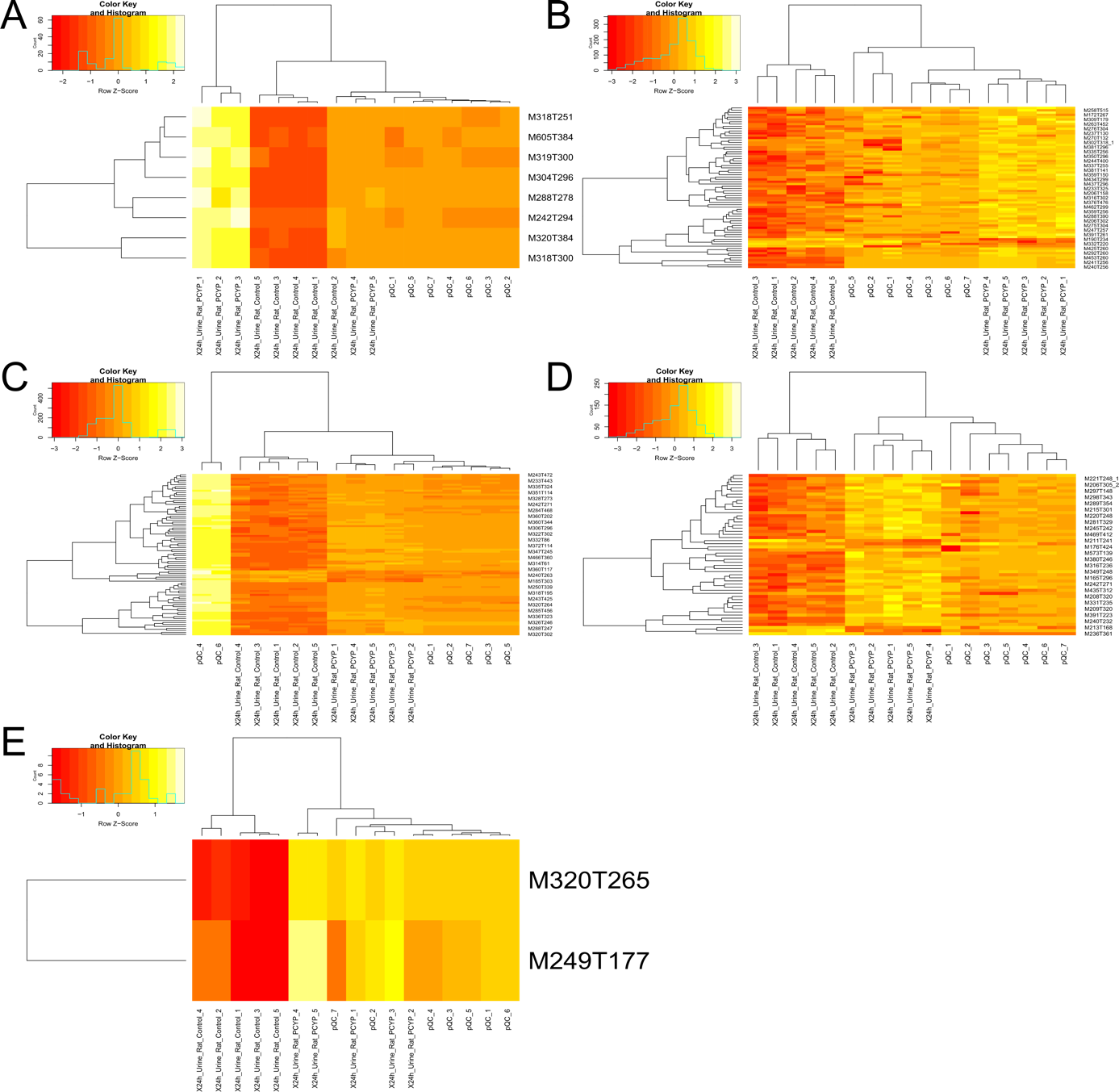


**Figure S12.** Results of heat map of hierarchical clustering of rat urine samples after analysis using hydrophilic interaction chromatography in positive (pos) and negative (neg) ionization mode. A = Nucleodur pos, B = Nucleodur neg, C = ZicHILIC pos, D = ZicHILIC neg, and E = PGC pos.


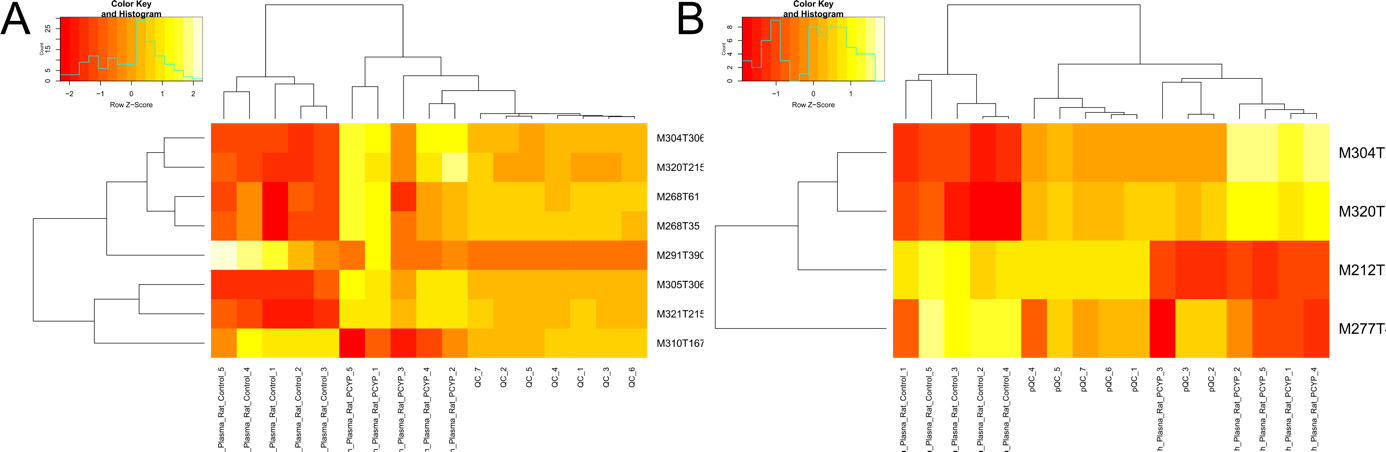


**Figure S13.** Results of heat map of hierarchical clustering of rat plasma samples after analysis using reversed-phase chromatography in positive ionization mode. A = Phenyl-hexyl and B = BEH.


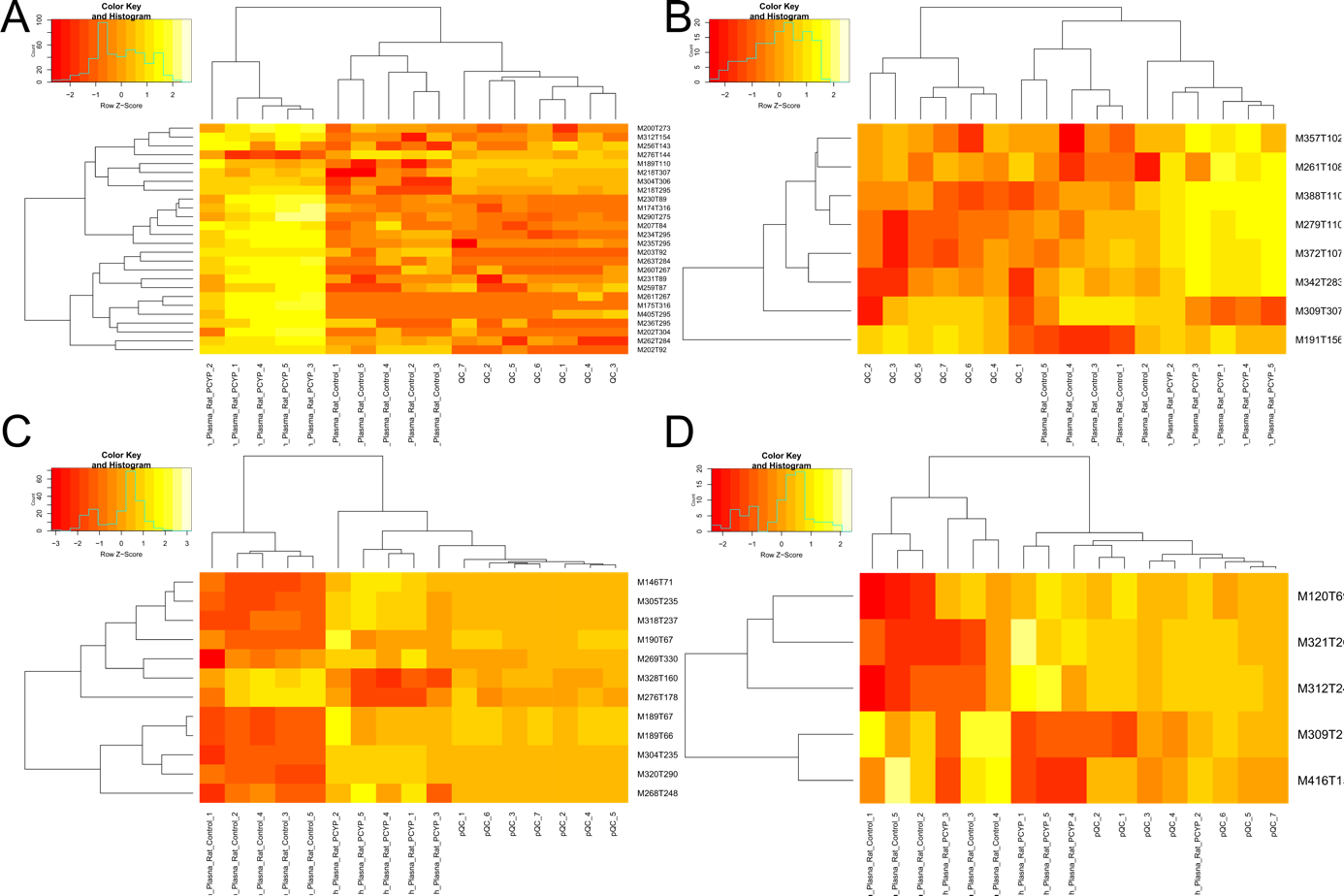


**Figure S14.** Results of heat map of hierarchical clustering of rat plasma samples after analysis using hydrophilic interaction chromatography in positive and negative ionization mode. A = Nucleodur pos, B = Nucleodur neg, C = ZicHILIC pos, and D = ZicHILIC neg.

# References

Adusumilli, R. and Mallick, P. (2017) Data Conversion with ProteoWizard msConvert. *Methods Mol Biol* **1550,** 339-368.

Hemmer, S., Manier, S.K., Fischmann, S., Westphal, F., Wagmann, L. and Meyer, M.R. (2020) Comparison of Three Untargeted Data Processing Workflows for Evaluating LC-HRMS Metabolomics Data. *Metabolites* **10**.

Hemmer, S., Wagmann, L. and Meyer, M.R. (2021) Altered metabolic pathways elucidated via untargeted in vivo toxicometabolomics in rat urine and plasma samples collected after controlled application of a human equivalent amphetamine dose. *Arch Toxicol* **95,** 3223-3234.

Hemmer, S., Wagmann, L., Pulver, B., Westphal, F. and Meyer, M.R. (2022) In Vitro and In Vivo Toxicometabolomics of the Synthetic Cathinone PCYP Studied by Means of LC-HRMS/MS. *Metabolites* **12**.

Manier, S.K., Wagmann, L., Flockerzi, V. and Meyer, M.R. (2020) Toxicometabolomics of the new psychoactive substances alpha-PBP and alpha-PEP studied in HepaRG cell incubates by means of untargeted metabolomics revealed unexpected amino acid adducts. *Arch Toxicol* **94,** 2047-2059.

Sumner, L.W., Amberg, A., Barrett, D., Beale, M.H., Beger, R., Daykin, C.A., Fan, T.W., Fiehn, O., Goodacre, R., Griffin, J.L., Hankemeier, T., Hardy, N., Harnly, J., Higashi, R., Kopka, J., Lane, A.N., Lindon, J.C., Marriott, P., Nicholls, A.W., Reily, M.D., Thaden, J.J. and Viant, M.R. (2007) Proposed minimum reporting standards for chemical analysis Chemical Analysis Working Group (CAWG) Metabolomics Standards Initiative (MSI). *Metabolomics* **3,** 211-221.
